# The Effects of Doxorubicin-based Chemotherapy and Omega-3 Supplementation on Mouse Brain Lipids

**DOI:** 10.3390/metabo9100208

**Published:** 2019-09-29

**Authors:** Djawed Bennouna, Melissa Solano, Tonya S. Orchard, A. Courtney DeVries, Maryam Lustberg, Rachel E. Kopec

**Affiliations:** 1 Department of Human Sciences, Human Nutrition Program, The Ohio State University, Columbus, OH 43210, USA; bennouna.1@osu.edu (D.B.); solano.20@osu.edu (M.S.); orchard.6@osu.edu (T.S.O.); 2 Neuroscience, College of Medicine, Columbus, OH 43210, USA; courtney.devries@hsc.wvu.edu; 3 Rockefeller Neuroscience Institute, West Virginia University, Morgantown, WV 26506, USA; 4 College of Medicine, Comprehensive Cancer Center, The Ohio State University Wexner Medical Center, Columbus, OH 43210, USA; maryam.lustberg@osumc.edu; 5 Foods for Health Discovery Theme, The Ohio State University, Columbus, OH 43210, USA

**Keywords:** specialized pro-resolving mediators, hippocampus, EPA, DHA, lipidomics, chromatography, mass spectrometry

## Abstract

Chemotherapy-induced cognitive impairment affects ~30% of breast cancer survivors, but the effects on how chemotherapy impacts brain lipids, and how omega-3 polyunsaturated fatty acid supplementation may confer protection, is unknown. Ovariectomized mice were randomized to two rounds of injections of doxorubicin + cyclophosphamide or vehicle after consuming a diet supplemented with 2% or 0% EPA+DHA, and sacrificed 4, 7, and 14 days after the last injection (study 1, *n* = 120) or sacrificed 10 days after the last injection (study 2, *n* = 40). Study 1 whole brain samples were extracted and analyzed by UHPLC-MS/MS to quantify specialized pro-resolving mediators (SPMs). Lipidomics analyses were performed on hippocampal extracts from study 2 to determine changes in the brain lipidome. Study 1 results: only resolvin D1 was present in all samples, but no differences in concentration were observed (*P* > 0.05). Study 2 results: chemotherapy was positively correlated with omega-9 fatty acids, and EPA+DHA supplementation helped to maintain levels of plasmalogens. No statistically significant chemotherapy*diet effect was observed. Results demonstrate a limited role of SPMs in the brain post-chemotherapy, but a significant alteration of hippocampal lipids previously associated with other models of cognitive impairment (i.e., Alzheimer’s and Parkinson’s disease).

## 1. Introduction

Breast cancer is the most commonly diagnosed cancer in women worldwide [1], with ~2 million new cases diagnosed and 627,000 deaths reported in 2018 [2]. Chemotherapy treatment has contributed to increased survival rates for millions of women [3], and due to its wide spectrum of activity, doxorubicin (DOX) is one of the most common agents prescribed alone or in combination with other antineoplastic drugs [3]. However, a growing body of evidence suggests that these drugs can be a double-edge sword, with chemotherapy-induced cognitive impairment (CICI) accompanying increased survival rates [4]. Indeed, nearly 30% of breast cancer survivors continue to experience chronic cognitive impairment 1–10 years after treatment [5,6,7], reflected in verbal fluency deficits, reduced working memory, reduced cognitive processing speed, increased depression, and increased anxiety [8,9].

One of the hypothesized mechanisms by which DOX-based chemotherapy may negatively impact cognition is via inflammation. Indeed, an increase in the expression of the pro-inflammatory cytokine IL-1b was observed in the cortex and hippocampus of ovariectomized mice at 7 days after DOX treatment [10]. Likewise, other work has reported increases in TNF-α in the cortex and hippocampus of DOX-treated mice [11]. However, because DOX does not cross the blood–brain barrier [11,12], inflammatory effects must be indirect. Previous studies have suggested that inflammation could reach the brain via several pathways, to stimulate microglial cells to produce inflammatory cytokines [11,13]. Other studies suggest that an increase in TNF-α enhances the expression of inducible nitric oxide synthase (iNOS), potentially leading to the nitration of proteins, such as manganese superoxide dismutase (MnSOD). This nitration could consequently decrease MnSOD activity and lead to an increase in reactive oxygen species (ROS) [14], which in turn can damage unsaturated brain lipids [15].

Therefore, decreasing brain inflammation may be an effective strategy to attenuate CICI observed after DOX treatment. Because of their well-established roles as precursors of anti-inflammatory signaling molecules in the inflammatory cascade [16] long-chain omega-3 polyunsaturated fatty acids, like eicosapentaenoic acid (EPA) and docosahexaenoic acid (DHA), have been pursued to attenuate a somatic inflammatory response. A double-blind placebo-controlled study of EPA + DHA supplementation in lung cancer patients actively undergoing chemotherapy demonstrated a significant decrease in plasma C-reactive protein (CRP) and interleukin-6 concentrations in the supplemented group after 66 days, relative to controls [17]. Likewise, higher concentrations of CRP are associated with greater levels of fatigue, and a higher omega-6 to omega-3 intake ratio, in breast cancer survivors [18]. Thus, it is possible that EPA+DHA treatment could attenuate CICI. This hypothesis is supported by previous work in DOX-treated mice, where animals concurrently supplemented with 2% DHA + EPA had increased *Shank3* gene expression (which codes for a protein involved in synapse formation), as compared to animals receiving no supplementation [10]. While the anti-inflammatory properties of EPA and DHA in somatic tissues are at least partially driven by their metabolites, specialized pro-resolving lipid mediators (SPMs) [16], little is known regarding the role of SPMs in the resolution of inflammation in the brain.

The main goal of this study was to investigate the impact of DOX chemotherapy on the brain lipidome, and the potential protective effect of DHA+EPA supplementation, on brain lipids. An ovariectomized mouse model was used because the majority of women undergoing chemotherapy treatment for breast cancer are postmenopausal [10,19]. Given that estrogen can have powerful pro-resolving properties [20], particularly in the brain [21], this model may be expected to mimic the more inflammatory state noted in women following menopause [22]. Whole brain SPMs were quantitated using targeted UHPLC-MS/MS (study 1) and the impact of DOX-chemotherapy and DHA+EPA supplementation on the center of memory and learning, i.e., the hippocampus, was assessed via untargeted UHPLC-MS analyses (study 2).

## 2. Materials and Methods

### 2.1. Animal Experiments and Diets

This study was conducted in accordance with the National Institutes of Health Guide for the Care and Use of Laboratory Animals, and under protocol #2015A00000040 approved by the Ohio State Institutional Animal Care and Use Committee. Mice were randomly assigned to diet and treatment groups, and all data were collected by individuals who were blinded to the experimental assignment. Full details of the animal studies can be found in Orchard et al. [10].

#### 2.1.1. Study 1

One week after ovariectomy, mice were randomized to a diet supplemented with 2% EPA + DHA supplementation (*n* = 60), or a diet with 0% EPA + DHA supplementation (*n* = 60), incorporated into an AIN-76A diet with low sucrose, prepared by Research Diets, Inc, Newark, NJ [10]. A dose of 2% of kcal as EPA + DHA was chosen because of preliminary data from our pilot study in postmenopausal breast cancer survivors suggesting that this dose reduced serum interleukin 6 levels in women who had chemotherapy and adjuvant aromatase inhibitor treatment [23]. Two and four weeks after beginning the diet, the animals received a total of two tail vein injections of either a cocktail of DOX (9 mg/kg) + cyclophosphamide (90 mg/kg), or sterile isotonic saline vehicle. Bodyweight was recorded prior to each injection, and the chemotherapy dose was calculated to be 50% of the typical human dose, based on body surface area [24]. Mice were sacrificed and whole brain was collected at 4, 7, and 14 days after the second injection, and snap frozen with liquid N_2_, to determine changes in SPMs and the time course of inflammation and resolution of inflammation. Samples were stored at −80 °C.

#### 2.1.2. Study 2

As in Study 1, one week after ovariectomy, mice were randomized to one of four diets (2% EPA + DHA or 0% EPA + DHA supplementation, with low sucrose or high sucrose content), and the injection regimen followed as described above [10]. Only animals receiving the low sucrose diet in combination with 2% EPA + DHA (*n* = 20) or 0% EPA+DHA supplementation (*n* = 20) were considered in the present work. Animals were sacrificed 10 days post-chemotherapy to facilitate behavioral testing on days 4–10 after the second injection [10]. Hippocampal tissues were isolated, and snap frozen with liquid N_2,_ to evaluate the changes in the hippocampal lipidome.

### 2.2. Chemicals

Optima grade methanol (MeOH), acetonitrile (ACN), isopropanol (ISP), and acetic acid (AcOH, > 98% pure) were purchased from Fisher Scientific (Pittsburg, PA, USA). UHPLC-grade methyl tert-butyl ether (MTBE) and hexane were also purchased from Fisher Scientific (Pittsburg, PA, USA). Methyl formate (> 98% pure) was purchased from Acros Organics (Morris Plains, NJ, USA). Ammonium acetate (NH_4_OAc) was purchased from Fisher Scientific (Fair Lawn, NJ, USA). Deionized water was obtained through filtration via a Millipore Q-Plus (Elix^®^35, Molsheim, France). For study 1, native SPM standards: resolvin D1 (RvD1), resolving D2 (RvD2), resolving D3 (RvD3), resolvin D5 (RvD5), resolving E1 (RvE1), maresin (MaR1), and protectin (PD1), and deuterated SPM standards: resolvins (RvD1-d_5_, RvD2-d_5_, RvD3-d_5_, RvE1-d_4_) maresin (MaR1-d_5_), and leukotriene B4-d_4_ were purchased from Cayman Chemicals (Ann Arbor, MI, USA). C18 SPE cartridges (Supel^TM^-Select HLB SPE 30 mg loading, 1 mL volume) were purchased from Supelco Analytical (Bellefonte, PA, USA). For study 2, lipid standards were purchased from Cayman Chemical (Ann Arbor, MI, USA), Santa cruz Biotechnology, Inc (Dallas, TX, USA) and Avanti polar Lipids, Inc (Alabaster, AL, USA), as summarized in Supplementary Materials: Table S1.

### 2.3. Targeted SPM Analysis (Study 1)

#### 2.3.1. Brain Tissue Extraction

The extraction protocol was adapted from a previously described method [25]. Briefly, whole brain tissue (50 mg) mixed with MeOH (0.5 mL), 10 μL of the deuterated SPM cocktail, and zircona beads (0.5 mm). Samples were ground using a Mini-bead beater-16 (Biospec Products, Tulsa, OK, USA) for 5 min., and centrifuged for 10 min (4 °C, 10,000 rpm) using a Microfuge 22R Centrifuge (Beckman Coulter, Brea, CA, USA). The supernatant was transferred to a fresh tube and centrifuged again. The supernatant was dried under N_2_ to ~70 μL, then acidified with 650 µL of deionized H_2_O acidified to pH 3.5 with hydrochloric acid. The samples were loaded onto C18 SPE cartridges preconditioned with MeOH (200 μL) and distilled water (400 μL), placed on a vacuum manifold (Resprep^®^ 12-port vacuum manifold system, Restek, Bellefonte, PA, USA). Samples were then flushed with deionized H_2_O (300 μL), hexane (360 μL), and SPMs eluted with methyl formate (720 μL). The extracts in methyl formate were dried under N_2_ gas, and stored at −80 °C no more than 24 h before analysis.

#### 2.3.2. UHPLC-MS/MS Analyses

Extracts were reconstituted in 150 μL MeOH/deionized H_2_O (1:1, *v*/*v*) and separated on a C18 column (Eclipse Plus, Agilent Technologies, Santa Clara, CA, 4.6 mm × 100 mm, 1.8 μm particle size) on an Ultimate 3000 UHPLC (Thermo Scientific, Waltham, MA, USA), coupled with an TSQ Quantiva triple quadrupole mass spectrometer, with an ESI probe operated in negative ion mode. The chromatographic separation was modified from a previous protocol [25] using a binary system of solvent A (0.1% AcOH in water, *v*/*v*) and solvent B (0.1% AcOH in MeOH, *v*/*v*) with the following gradient: held at 55% B from 0 to 2 min, linear increase to 85% B over 8 min, linear increase to 90% B over 3 min, holding at 98% B for 2 min, and rapidly returning to 55% B and holding for 7 min. Sample injection volume was 15 μL, flow rate was 0.4 mL/min, and the column was held at 40 °C. The MS source parameters of analysis were: spray voltage = −2000 V, ion transfer tube temperature = 300 °C, vaporizer temperature = 325 °C, sheath gas flow = 45 (arbitrary units), aux gas flow = 13 (arbitrary units), sweep gas flow = 1 (arbitrary units). Previously reported transitions unique to native SPMs and their deuterated counterparts [25] were verified, and collision energies tested for optimal performance on this instrument. Analytes were followed via multiple reaction monitoring (MRM) with a cycle time of 0.6 s, and peak areas of both native and deuterated species, in conjunction with external calibration curves, used for quantitation. Additional details (i.e., precursor ions, qualitative product ions, quantitative product ions, and collision energies) are provided in Table S2.

#### 2.3.3. Statistical Analysis

R software version 3.5.2 was used for analyses [26] with package Rcmdr version 2.5-2 [27], to verify that RvD1 concentrations met the assumptions of normality and variance. A linear model using the lme4 package [28] was constructed to determine the effect of the main factors of diet, chemotherapy, and sacrifice day on RvD1 concentrations, as well as the interaction of diet*chemotherapy*sacrifice day. The likelihood ratio test was used to determine the significance of any one effect on the full model, with (*P* < 0.05) considered statistically significant.

### 2.4. Untargeted Lipidomics (Study 2)

#### 2.4.1. Hippocampal Tissue Extraction

The extraction protocol was adapted from a previously described method [29]. Hippocampal weights (~10 mg) were measured and the tissue mixed with methanol/MTBE (400 μL, 1:3, *v*/*v*) before homogenization (PowerGen 500, Fisher Scientific, San Diego, CA, USA). Samples were then probe sonicated for 20 s, 2 Watts (Misonix, Farmingdale, NY, USA), and shaken (Rocking Platform Shaker, VWR International, USA) for 20 min at 4 °C. Afterward, a methanol/water mixture (400 μL, 1:3, *v*/*v*) was added, and samples were vortexed for 10 s and centrifuged for 7 min (4 °C, 14,000 rpm), using a Microfuge 22R Centrifuge (Beckman Coulter, Brea, CA). Supernatant (150 μL) was collected, dried under argon, and stored at −80 °C until analysis.

#### 2.4.2. UHPLC-MS Analyses

Extracts were reconstituted in ACN/ISP (150 μL, 7:3, *v*/*v*), followed by 20 s of probe sonication (Misonix, Farmingdale, NY, USA) and 5 min centrifugation (4 °C, 14,000 rpm) before analysis with an Agilent 1290 UHPLC coupled to an Agilent 6545 quadrupole time-of-flight mass spectrometer (Agilent Technologies, Santa Clara, CA, USA). Chromatographic separation was performed using a previously published reverse phase method employing a C8 column (Acquity Plus BEH, Waters, Milford, MA, 100 mm × 2.1 mm, 1.7 μm particle size) and an H_2_O/ACN/ISP mobile phase with NH_4_OAc and acetic acid modifiers [29]. The injection volume was 5 μL. The following ionization source parameters were utilized: spray voltage negative ion = −2000 V, ion transfer tube temperature = 300 °C, vaporizer temperature = 325 °C, sheath gas flow = 45 (arbitrary units), aux gas flow = 13 (arbitrary units), sweep gas flow = 1 (arbitrary units). The mass spectrometer was tuned and calibrated before the analysis, and the manufacturer’s reference mixture analyzed throughout every sample injection every second scan for real-time-of-flight calibration (as performed by the software). Pooled quality control samples (QC) were analyzed every 6^th^ injection, as well as process blanks ~25^th^ injection, to correct for instrument performance and remove persistent contaminant features from the data, respectively.

#### 2.4.3. Data Processing

Raw LC-MS data was collected using Agilent MassHunter (version B.08.00), and processed using Agilent Profinder (version 10.0). Batch recursive feature extraction was used to deconvolute and align the molecular features in all samples, using a feature extraction cut-off of peak height ≥ 1000 counts, charge state = 0, 1, or 2. For binning and alignment, a retention time tolerance of 0.1 min and a 5 ppm cutoff was set. Samples were normalized to wet weight, and post-processing filters applied to eliminate features with: height < 2000 counts, features in the process blank, features present in < 80% of the QC samples, and features with ≥ 30% relative standard deviation.

### 2.5. Statistical Analysis

Data intensities tables were exported from the Agilent software, and Simca-P (version 14, Sartorius Stedim Biotech, Umeå, Sweden) used to perform principal component analysis (PCA), and partial least squares-discriminant analysis (PLS-DA). The statistical significance of the PLS-DA classification model was assessed using a permutation test (200 permutations) and cross-validation–analysis of variance (CV-ANOVA). The predictive capacity of the model was evaluated by the *R^2^* and *Q^2^*. *T*-tests were performed to determine the significance of each individual metabolite with an FDR-corrected *P*-value < 0.05 considered statistically significant, using MetaboAnalyst [30]. The interaction between chemotherapy treatment and DHA+EPA supplementation was evaluated both using a two-way ANOVA and using a mixed model [28], using the R packages described previously.

### 2.6. Compound Identification

Identification of the discriminating variables was performed by fragmenting the candidate ion by ramping collision energy from 10–40 V, and comparing the resulting MS/MS spectra with those in databases (i.e., LipidBlast, LIPID MAPS), as searched by MS-Dial [31] and MS-Finder [32]. Authentic lipid standards were then analyzed in conjunction with candidate metabolites to confirm retention time, precursor ion, and fragmentation patterns (when sufficient precursor signal was present to observe distinguishable product ions). Metabolite identification levels are reported according to the definitions set forth by the Metabolite Identification Task Group [33].

## 3. Results

### 3.1. SPM Detection and Quantification in Whole Brain Extract

**Figure 1:**
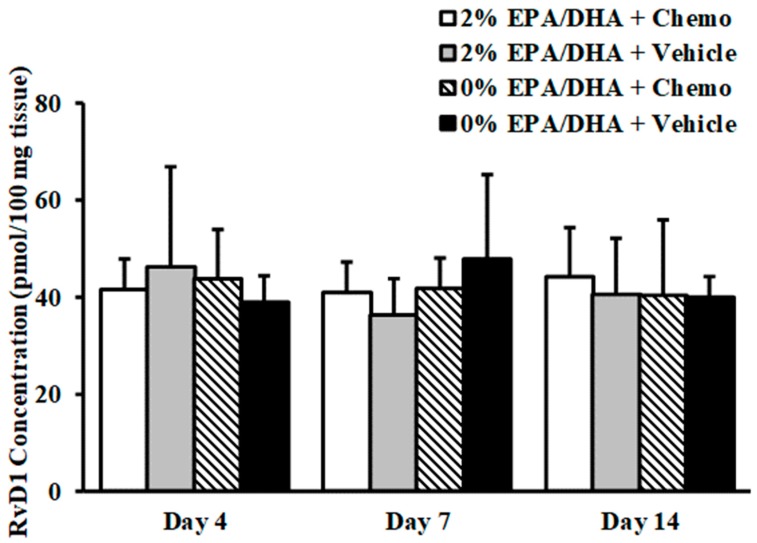
Concentrations of RvD1 in brain tissue extract in animals sacrificed 4, 7, and 14 days after the last chemotherapy or vehicle treatment, (*n* = 8–12 for each group) represented as means ± standard error of the mean. There was no main effect of diet supplementation, chemotherapy group, nor day on RvD1 levels (*P* > 0.05). Chemo = DOX (9 mg/kg) + cyclophosphamide (90 mg/kg) treatment.

Extraction recoveries were between 88–104%. RvD1 was consistently observed in all whole brain extracts tested (Figure 1). However, no significant influence of the main effects of diet, chemotherapy, or day of sacrifice were observed on RvD1 tissue concentrations, nor the interaction of diet*chemotherapy*day of sacrifice (*P* > 0.10). Three additional SPMs, i.e., RvD3, MaR1, and PD1, were detected in the whole brain tissue of a handful of animals (with detection defined as a signal to noise ratio > 3 but < 10). These analytes were identified via precursor > product ions consistent with those of the external standards, and retention times which aligned with those of the stable isotope internal standards. RvD2, RvD5, and RvE1 were not observed in any of the extracts.

### 3.2. Untargeted Lipidomics

#### 3.2.1. Impact of Chemotherapy on Hippocampal Lipidome

The chromatographic conditions used in this study provided good peak resolution (Figure S1), with a total of 1016 metabolites detected. PCA plots of the individual samples before and after correction for pooled QC sample drift are presented in Figure S2, demonstrating alignment of the pooled QC samples (green points) post-processing, with 500 metabolites remaining. A PCA was performed with both treatment and diet groups, to visualize the distribution of all the samples. No clustering was observed (Figure S3).

**Figure 2:**
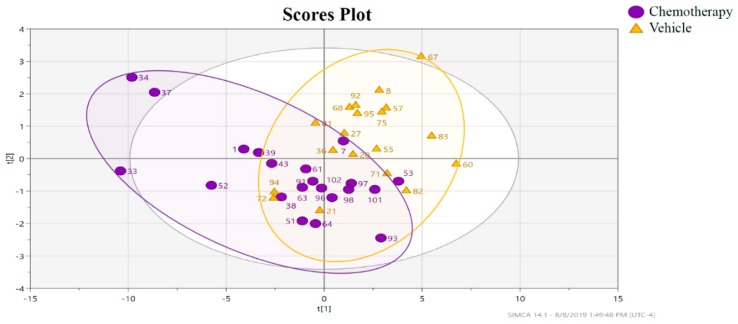
Two dimensional partial least squares-discriminant analysis (PLS-DA) plot with data points representing individual animals treated with chemotherapy (purple dot, *n* = 21) or vehicle (orange triangles, *n* = 19), after feature selection (26 metabolites with a VIP score > 1.75 for the first component were retained). *R^2^* = 71%, *Q^2^* = 0.275, CV-ANOVA *P* value = 0.01, and the post-permutation test (200 permutations) *Q^2^* value = −0.085.

**Figure 3:**
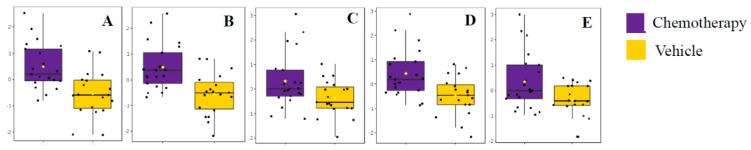
Box-plots demonstrating the median (central bar), 25% and 75% of metabolite intensities of the compounds impacted by chemotherapy, with *n* ≈ 20 per treatment group. (**A**) nervonic acid, (**B**) 13(*Z*)-docosenoic acid, (**C**) eicosatrienoic acid isomer, (**D**) 11(*Z*)-eicosenoic acid, (**E**)-linoleic acid.

PCA was then used to visualize the impact of chemotherapy alone on the hippocampal lipidome, by comparing the chemotherapy and vehicle groups of the 0% EPA + DHA supplemented animals. PCA explained 72.8% of the total variance in 3 principal components. Overall, no natural clustering was noted in the first and second PCA components (Figure S4), thus PLS-DA was used to elucidate metabolites that drove the chemotherapy effect. Only the analytes with a variable importance in projection (VIP) score > 1.75 were retained in the final model (26 metabolites). As shown in Figure 2, very significant class separation was observed among the chemotherapy and vehicle treatments (*R^2^* = 71%, *Q^2^* = 0.275, CV-ANOVA *P*-value = 0.01, post-permutation test *Q^2^* value = −0.085). Conclusive metabolite identification was performed for 12 of the 26 metabolites whose individual false discovery rate (FDR) adjusted *P*-value (*q*-value) < 0.05. Four of the metabolites were unequivocally confirmed with authentic standards as nervonic acid, 13(*Z*)-docosenoic acid, 11(*Z*)-eicosanoic acid, and linoleic acid. The metabolite with *m*/*z* 305 (predicted to be a precursor of [M−H]^−^), exhibited the same fragmentation pattern as the 5(*Z*),8(*Z*),11(*Z*)-eicosatrienoic acid standard (C20:3). However, the LC-MS analysis of the standard revealed a slightly later retention time relative to the metabolite in the hippocampal extract, and is presumed to be a geometrical isomer. All remaining analytes were either putatively classified into a metabolite family based on their exact mass (level 3) or marked as unknown (level 4, see Table 1). Nervonic acid, 13(*Z*)-docosenoic acid, 11(*Z*)-eicosanoic acid, linoleic acid, and the eicosatrienoic acid isomer were significantly more abundant in the hippocampus of animals that received a chemotherapy, as compared to those receiving the vehicle injection (Figure 3A–E).

#### 3.2.2. Impact of EPA + DHA Supplementation on the Hippocampal Lipidome

**Figure 4:**
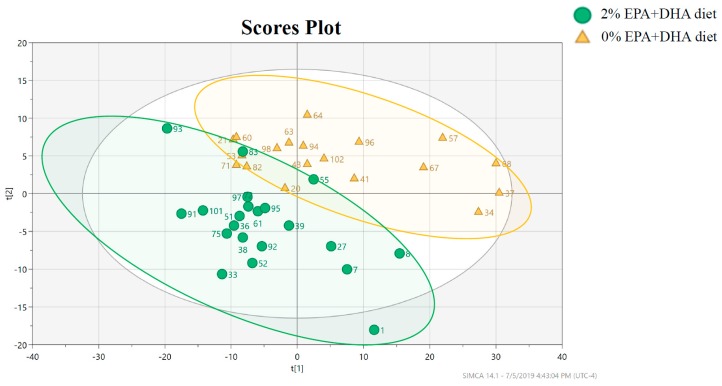
Two dimensional PLS-DA plot with data points representing individual animals supplemented with 2% EPA+DHA diet (green dot, *n* = 21) or 0% EPA+DHA diet (orange triangles, *n* = 19) performed on 500 analytes. *R^2^* = 63%, *Q^2^* = 0.553, CV-ANOVA *P* value = 5.98 × 10^−7^, and the post-permutation test (200 permutation) *Q^2^* = −0.072).

Comparisons were made between the animals consuming 0% EPA+DHA supplemented diet vs. animals consuming the 2% EPA+DHA diet. No natural clustering was observed from the PCA when selecting the first and second components, which explained 66.4% of the total observed variance (Figure S5). PLS-DA was then performed on all 500 of the detected analytes, to better visualize the EPA+DHA effect (Figure 4). Very significant class separation was observed between the two groups (*R^2^* = 63%, *Q^2^* = 0.553, CV-ANOVA *P* value = 5.98*10^−7^, post-permutation *Q^2^* = −0.072). *T*-tests (FDR < 0.05) revealed that 15 metabolites were significantly impacted by EPA+DHA supplementation (Table 2).

**Figure 5:**
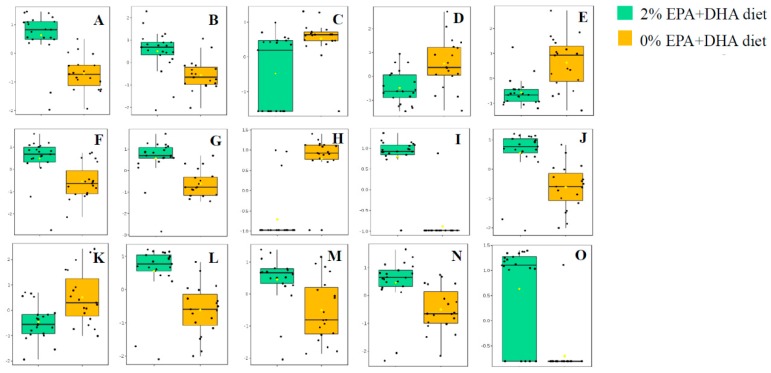
Box-plots demonstrating the median (central bar), 25% and 75% metabolite intensities of the compounds impacted by omega-3 supplementation, with *n* ≈ 20 per supplementation group. (**A**) eicosapentanoic acid, (**B**) docosapentanoic acid, (**C**) eicosadienoic acid, (**D**) PG 20:4/22:6, (**E**) PG 18:2/18:2, (**F**) PC 16:0/18:3, (**G**) PC 16:0/20:5, (**H**) PC 16:0/18:2, (**I**) PE 16:1/20:4, (**J**) PE 16:0/22:5, (**K**) PE 18:1/20:3, (**L**) PE (P-16:0/20:5), (**M**) PE (P-18:0/22:6), (**N**) PE 22:6/22:5, (**O**) PI 18:0/20:5.

The identification of 3 metabolites, EPA, docosapentanoic acid (DPA), and eicosadienoic acid (EDA), were unequivocally confirmed with authentic standards. EPA and DPA were more abundant in the group consuming a 2% EPA+DHA supplemented diet (Figure 5A,B), while EDA was more abundant in the group consuming a 0% EPA+DHA supplemented diet (Figure 5C). All 12 remaining metabolites were identified as glycerophospholipids (glycerophosphoglycerols (PG), glycerophosphocholines (PC), and glycerophosphoethanolamines (PE)) based on their distinctive fragmentation patterns. Details of identification are provided in the Online Supplemental Information (Figures S6–S11). Animals consuming the 2% EPA + DHA supplemented diet had higher levels of PI 18:0/20:5, PE (P-18:0/22:6), PE (P-16:0/20:5), PE 22:6/22:5, PE 16:0/22:5, PE 16:1/20:4, PC 16:0/18:3, PC 16:0/20:5, and lower levels of PG 20:4/22:6, PG 18:2/18:2, PC 16:0/18:2 and PE 18:1/20:3 relative to animals consuming the 0% EPA + DHA supplemented diet (Figure 5D–O).

It should be noted that two of these metabolites were observed in the 2% EPA + DHA supplement: EPA and DPA. The presence of ions in the diet whose *m*/*z* values were consistent with those of PE (P-16:0/20:5) and PE (P-18:0/22:6) were also observed, although the retention times did not align with those measured in the hippocampal brain extracts, suggesting isomerization. Alternatively, the fact that phospholipids are cleaved during digestion before absorption [34] leaves open the possibility that these hippocampal phospholipids may have been endogenously biosynthesized.

#### 3.2.3. Interaction between the Impact of Chemotherapy and of Omega-3 Supplementation

The results obtained from the two-way ANOVA and the mixed linear model revealed no significant interaction effect between chemotherapy group*omega-3 supplementation group, as further visualized in Figure S12.

## 4. Discussion

To the best of our knowledge, this is the first report studying the effects of chemotherapy on the resolution of brain inflammation. The SPM RvD1 was quantitated in all whole brain extracts, but no significant difference in concentration was observed (regardless of chemotherapy or diet group). The remaining SPMs were either not observed at all, or only observed in a few extracts tested. This is also the first report of the effects of chemotherapy and dietary EPA + DHA supplementation on the lipidome of the hippocampus, a key region involved in learning and memory [35].

The anti-inflammatory effects of EPA + DHA are at least partially mediated via an active process driven by SPMs. SPMs are metabolites enzymatically derived from EPA + DHA via modification by lipoxygenase [36] after an acute inflammatory event. Although well established in peripheral tissues [16], this active process of inflammation resolution has not convincingly been demonstrated in the brain.

While RvD1 was observed in all tissues, no differences were observed between concentrations. This result is in agreement with a study of SAMP8 animals, a murine model of accelerated aging displaying cognitive decline [37]. Despite a greater degree of inflammation in SAMP8 mice over 9 months, no commensurate increase in RvD1 or other measured SPMs, such as Lipoxin A4, were observed [37].

The detection of RvD3, MaR1, and PD1 in only a few animals, and an absence of RvD2, RvD5, and RvE1 suggest either that concentrations of these SPMs were below limits of method detection (~1.5-fold higher than the LOD previously reported for blood [25]), matrix suppression, or that they are absent from the brain tissue altogether. Similarly, Hopperton et al. reported no detectable concentrations of the SPMs protectin, maresin or resolvins (D and E series) in Fat-1 mouse model of Alzheimer’s disease (AD), and these concentrations remained undetectable over 12 weeks, despite consumption of a diet containing 2.4% EPA and 1.1% of DHA [38]. Trépanier et al. [39] also noted a general absence of SPMs following direct intracerebral ventricular injection of LPS (a bacterial metabolite which stimulates a measureable inflammatory response) in a murine model over 28 days, causing the authors to conclude that the resolution of brain inflammation was mediated independently of SPMs.

In contrast, Orr et al. reported PD1, 17-hydroxy DHA, RvD5, and MaR1 (and no detectible levels of RvD1 or RvD2) in mouse hippocampus after delivering an LPS injection [40]. During this study, the mice received a higher dose of LPS (5 μg/μL) compared to the dose used by Trépanier et al. (1 μg/μL) [39], which could explain the differences observed between the two studies. Belayev et al., also reported that DHA supplementation potentiated neuroprotectin D1 (NPD1) synthesis 3 days after a stroke-inducing cerebral artery occlusion in a murine model [41].

The disparity between these results are challenging to reconcile, but it should be noted that one of the studies reporting measurable brain SPM concentrations also reported neutrophil infiltration [42], which carry the lipoxygenase enzymes that synthesizes SPMs. Similarly, the studies reporting a lack of SPMs [39,43] do not report on infiltrating neutrophils. Thus, an absence of neutrophil infiltration may also explain the lack of SPMs reported herein. Lastly, although a reduction in estrogen (which occurs in an ovariectomized model) increases the inflammatory response [20], the reduction in estrogen may have also attenuated the concentration of SPMs produced [21].

The hippocampus is a major component of the brain of humans and other vertebrates [35] and is one of the regions containing the highest levels of lipids [44], especially glycerophospholipids [45]. Moreover, the hippocampus has been studied extensively as part of the brain system responsible for spatial memory and navigation, and is considered to be the first region of the brain to suffer damage in cognitive disorders such as Alzheimer’s disease (AD) [46], epilepsy [47] and schizophrenia [48]. Because several studies have shown that most cognitive impairment is correlated with phospholipids expression level [49], and these species are better ionized in negative mode [50], we chose this approach for analyses.

Chemotherapy had a measurable impact on hippocampal lipids. Notably, chemotherapy treatment was associated with significantly higher concentrations of four metabolites involved in the omega-9 polyunsaturated fatty acid biosynthesis pathway (Figure S13), including nervonic acid, 13(*Z*)-docosanoic acid, 11(*Z*)-eicosenoic acid, and 5(*Z*),8(*Z*),11(*Z*)-eicosatrionic acid. Nervonic acid is particularly abundant in mammalian cerebral tissue [51] where it forms nervonyl sphingolipids which are enriched in the myelin fraction of myelinated nerve fibers [52]. Significant increases of nervonic acid, 13(*Z*)-docosanoic acid, 11(*Z*)-eicosenoic acid, and 5(*Z*),8(*Z*),11(*Z*)-eicosatrionic acid have been reported in the mid-frontal, hippocampal, and temporal cortexes of AD patients relative to age-matched controls [53].

Nasaruddin et al., reported higher concentrations of 13(*Z*)-docosanoic acid in the post-mortem neocortical tissue of late-stage AD subjects relative to age-matched controls [54]. A more recent study by Nasaruddin et al. reported that nervonic acid and 13(*Z*)-docosanoic acid were significantly higher in the post-mortem parietal cortex of moderate-AD vs. controls [55].

Increases in these omega-9 fatty acids have also been observed in the blood plasma of those suffering from cognitive impairment. Evidence from Iuliano et al., reported increased concentrations of 13(*Z*)-docosanoic acid and a step-wise increase in 5(*Z*),8(*Z*),11(*Z*)-eicosatrionic acid in the plasma of patients diagnosed with mild cognitive impairment or mild AD relative to healthy controls [56]. Likewise, Kageyama et al. reported increased blood plasma levels of nervonic acids in patients with major depressive disorder, relative to disease-free controls [57]. Thus, these specie(s) might serve as appropriate blood biomarker(s) to assess cognitive impairment, including that induced via chemotherapy. Furthermore, results presented herein suggest that increased flux through the omega-9 biosynthesis pathway, potentially via increases in stearoyl-CoA desaturase biosynthesis [53] or via increased demyelination [58], could be targets for reducing chemotherapy-induced brain damage.

A significant main EPA + DHA dietary supplementation effect was observed on the hippocampal lipidome. EPA and DPA were more abundant in the 2% EPA + DHA supplemented group relative to 0% EPA + DHA, likely due to the fact that the supplemented diet contained EPA and DPA, which plausibly accumulated in the tissue over several weeks of feeding. Several studies have reported the positive effects of EPA and DPA on memory and cognition. Kelly et al. found that DPA and EPA possessed neurorestorative effects and were capable of downregulating microglial activation in aged rats [59]. EPA has also been demonstrated to protect aged rats from increases in hippocampal IL-1β induced by amyloid-β (Aβ) oligomers [60], a causative agent of AD [61]. All the other lipids impacted by EPA + DHA supplementation were identified as glycerophopspholipids (PL), key components of cell lipid bilayers, which are also involved in cell metabolism and signaling. Results reveal six of the identified phospholipids highly abundant in the EPA + DHA supplemented group were omega-3-PL, with an omega-3 FA in the sn-2 position, i.e., PI 18:0/20:5, PE (P-18:0/22:6), PE (P-16:0/20:5), PE 22:6/22:5, PE 16:0/22:5 and PC 16:0/20:5. These results are in agreement with other studies that demonstrated increased concentrations of several phospholipids after supplementation with omega-3 fatty acids [62,63]. Increase of omega-3-PL may influence the fluidity and permeability of the cell membranes, enhancing neuroprotective properties [64].

Multiple studies have reported an inverse relationship between plasmalogen concentrations and AD. Since the first study in 1995 [65] describing lower concentrations of ethanolamine plasmalogen (PlsEtns) in post-mortem brain samples of AD patients, the same trend has also been reported in cerebrospinal fluid [66], plasma, serum, and red blood cells of AD patients [67] relative to healthy controls. Decreased PlsEtns have also been observed in AD patients in sites of neurodegeneration such as the hippocampus, temporal cortex, and frontal cortex [68,69]. Interestingly, our results showed a significant increase of two ethanolamine plasmalogens, PE (P-18:0/22:6) and PE (P-16:0/20:5), in the EPA + DHA supplemented animals. Based on these results and the studies described above, over the long-term, EPA + DHA supplementation could contribute to reducing DOX-induced changes in PE concentrations.

Several PLs were increased in the non-supplemented animals vs. the 2% EPA + DHA supplemented animals, including PG 20:4/22:6, PG 18:2/18:2, PC 16:0/18:2 and PE 18:1/20:3. These species are characterized by the presence of DHA, linoleic acid, linoleic acid, and mead acid, in the sn-2 position, respectively. Surprisingly, PG 20:4/22:6 and PG 18:2/18:2 were also observed at higher concentrations in the hippocampal lipid extracts of non-supplemented animals, although previous studies have reported very low concentrations of PG in the brain relative to other PLs [70]. Collectively, the data support that an EPA+DHA supplemented diet plays an important role in maintaining the concentrations of omega-3 fatty-acid derived PLs. Ultimately, this could contribute to an improvement in brain lipid levels which counteract the lipid changes observed after DOX-based chemotherapy treatment.

Results revealed no interaction between chemotherapy treatment and the EPA + DHA supplementation. This observation may be due to the fact that the EPA+DHA supplementation was not long enough to restore some of the lipid modifications induced by chemotherapy, and a longer duration of feeding may reveal a more favorable shift.

## 5. Conclusions

In conclusion, the results of the targeted SPM analysis point to a limited role of SPMs in mediating a DOX-induced inflammatory response. Hippocampal lipid changes after DOX treatment revealed increases in lipids previously reported to be higher in models of cognitive impairment (including AD), especially those of the omega-9 synthesis pathway. The EPA + DHA supplementation also induced fluctuations in a number of lipids previously reported in supplemented animals to be protective. Further investigations are essential to increase the understanding of the negative cognitive outcomes associated with chemotherapy, and in identifying biomarker(s) that may be distinct for CICI. Ultimately, this information can help to determine appropriate dietary strategies for this population.

## Figures and Tables

**Table 1:** List of metabolites significantly impacted by chemotherapy treatment.

Compound	Precursor Ion *m*/*z*	Mass Error ^a^ *ppm*	Retention Time (min)	Precursor Ion Species	Product Ions *m*/*z*	*P*-Value	FDR (*q*-Value)	Identification Level
Nervonic Acid	365.3419	0.13	11.18	[M − H]^−^	-	0.0006	0.005	confirmed (level 1)
Eicosenoic Acid	309.2799	1.93	8.67	[M − H]^−^	-	0.0027	0.01	confirmed (level 1)
Linoleic Acid	279.2325	0.36	6.13	[M − H]^−^	-	0.0185	0.04	confirmed (level 1)
Docosenoic Acid	337.3109	0.89	10.00	[M − H]^−^	-	0.0003	0.005	confirmed (level 1)
Eicosatrienoic Acid Isomer	305.2485	1.64	6.68	[M − H]^−^	303, 261	0.0254	0.04	putative (level 2)
22-tricosenoic Acid Isomer	351.3267	1.14	10.60	[M − H]^−^	-	0.0006	0.005	putative (level 3)
Tetracosatetraeonic Acid	359.2951	0.28	8.49	[M − H]^−^	-	0.0044	0.01	putative (level 3)
Lysophosphatidic Acid Isomer	409.2358	0.73	5.78	[M − H]^−^	-	0.0285	0.04	putative (level 3)
PG (22:0/18:1)	669.5595	1.34	15.33	[M-C_6_H_10_O_5_-H]^−^	-	0.0053	0.01	putative (level 3)
Unknown-1	720.6507	-	15.00	[M − H]^−^	-	0.0093	0.02	unknown
Unknown-2	425.2095	-	5.78	[M − H]^−^	-	0.0129	0.03	unknown
Unknown-3	477.2027	-	7.23	[M − H]^−^	-	0.0291	0.04	unknown

Abbreviations: PG = phosphatidylglycerol; ^a^ mass error in parts per million (ppm) = ((theoretical *m*/*z* – empirically measured *m*/*z*)/theoretical *m*/*z*)*10^6^.

**Table 2:** List of metabolites significantly impacted by dietary supplementation.

Compound	Precursor Ion *m*/*z*	Mass Error ^a^ *ppm*	Retention Time (min)	Precursor Ion Species	Product Ions *m*/*z*	*P*-Value	FDR (*q*-Value)	Identification Level
Eicosapentanoic Acid	301.2171	1.33	5.10	[M − H]^−^	-	1.52 × 10^−6^	0.0002	confirmed (level 1)
Docosapentanoic Acid	329.2484	1.21	6.33	[M − H]^−^	-	0.0003	0.01	confirmed (level 1)
Eicosadienoic Acid	307.2638	0.32	7.60	[M − H]^−^	-	0.0007	0.03	confirmed (level 1)
PG 20:4/22:6	841.5026	0.83	10.00	[M − H]^−^	327; 303	0.0007	0.03	putative (level 2)
PG 18:2/18:2	769.5010	−1.16	10.32	[M − H]^−^	279	3.89 × 10^−5^	0.003	putative (level 2)
PI 18:0/20:5	883.5329	−0.79	11.39	[M − H]^−^	581; 419; 301; 283; 241	1.62 × 10^−6^	0.0002	putative (level 2)
PE 22:6/22:5	836.5238	0.96	11.41	[M − H]^−^	329; 283	0.0015	0.04	putative (level 2)
PC 16:0/20:5	838.5609	1.31	11.53	[M+CH3COO]^−^	764; 301; 255	3.98 × 10^−5^	0.003	putative (level 2)
PC 16:0/18:3	814.5164	−4.17	11.53	[M+CH3COO]^−^	740; 277; 255	0.0005	0.02	putative (level 2)
PC 16:0/18:2	792.5384	9.33	12.20	[M + Cl]^−^	742; 279; 255	1.18 × 10^−8^	2.95 × 10^−6^	putative (level 2)
PE 16:1/20:4	736.4922	0.68	11.70	[M − H]^−^	303; 253	3.46 × 10^−12^	1.73 × 10^−9^	putative (level 2)
PE (P-16:0/20:5)	720.4970	0.28	12.14	[M − H]^−^	436; 418; 301	5.42 × 10^−5^	0.003	putative (level 2)
PE 16:0/22:5	764.5229	−0.13	12.28	[M − H]^−^	329; 255	0.0004	0.02	putative (level 2)
PE (P-18:0/22:6)	774.5448	1.42	12.78	[M − H]^−^	464; 446; 327	0.0015	0.04	putative (level 2)
PE 18:1/20:3	766.5376	−1.30	12.85	[M − H]^−^	305; 281	0.0013	0.04	putative (level 2)

Abbreviations: PG = phosphatidylglycerol, PI = phosphatidylinositol, PC = phosphatidylcholine, PE = phosphatidylethanolamine; ^a^ mass error in parts per million (ppm) = ((theoretical *m*/*z* – empirically measured *m*/*z*)/theoretical *m*/*z*)*10^6^.

## Supplementary Materials

The following are available online at https://www.mdpi.com/2218-1989/9/10/208/s1, Figure S1: Total ion chromatogram (LC-MS) of hippocampus extract ionized in negative mode; Figure S2: PCA scores plots depicting individual samples (blue dots), and pooled quality control (QC) samples (green dots) of (A) raw data before any filtration, and (B) filtered data (i.e., after removal of blank signals and false chromatographic peaks); Figure S3: Product ion spectra of (A) PG (20:4/22:6) showing fragmentation of the precursor [M−H]^−^ m/z 841 and (B) PG (18:2/18:2) showing fragmentation of the precursor [M−H]^−^ at m/z 769; Figure S4: Product ion spectra of (A) PC (16:0/20:5) at m/z 838 corresponding to [M+CH_3_COO]^−^, (B) PC (16:0/18:3) at m/z 814 corresponding to [M+CH_3_COO]^−^ and (C) PC (16:0/18:2) at m/z 792 corresponding to [M+Cl]^−^; Figure S5: Product ion spectra of (A) PE (16:1/20:4) at m/z 736 corresponding to [M−H]^−^, (B) PE (18:1/20:3) at m/z 766 corresponding to [M−H]^−^, and (C) PE (16:0/22:5) at m/z 764 corresponding to [M−H]^−^; Figure S6: Product ion spectra of (A) plasmalogen PE(P-16:0/20:5) at m/z 736 corresponding to [M−H]^−^, and (B) plasmalogen PE(P-18:0/22:6) at m/z 774, corresponding to [M−H]^−^; Figure S7: Product ion spectra of PS (18:0/22:5) at m/z 836, corresponding to [M−H]^−^; Figure S8: Product ion spectra of PI 18:0/20:5 at m/z 883, corresponding to [M−H]^−^; Figure S9: Omega-9 polyunsaturated fatty acid biosynthesis pathway^3^; Table S1: List of purified fatty acids used for fatty acid identification; Table S2: Parameters for LC-MS/MS analysis of specialized pro-resolving mediators (SPMs) in negative mode.

## Abbreviations

**ACN** = acetonitrile, **AD** = Alzheimer’s disease, **CICI** = chemotherapy-induced cognitive impairment, **CRP** = C-reactive protein, **DHA** = docosahexaenoic acid, **DOX** = doxorubicin, **DPA** = docosapentanoic acid, **EDA** = eicosadienoic acid, **EPA** = eicosapentaenoic acid, **ESI** = electrospray ionization, **FDR** = false discovery rate, **IL** = interleukin, **ISP** = isopropanol, **LPS** = lipopolysaccharides, **MRM** = multiple reaction monitoring, **MS** = mass spectrometry, **MTBE** = methyl tert-butyl ether, **PC** = phosphatidylcholine, **PCA** = principle component analysis, **PE** = phosphatidylethanolamine, **PG** = phosphatidylglycerol, **PI** = phosphatidylinositol, **PL** = phospholipids, PlsEtns = ethanolamine plasmalogens, **PLS-DA** = partial least squares-discriminant analysis, **QC** = quality control, **ROS** = reactive oxygen species, **SPE** = solid phase extraction, **SPM** = specialized pro-resolving mediators, **TNF-α** = tumor necrosis factor-α, **UHPLC** = ultra-high-performance liquid chromatography, **UV** = unit variance, **VIP** = Variable importance in projection.

## References

[1] Amarante M.K., de Sousa Pereira N., Vitiello G.A.F., Watanabe M.A.E. (2019). Involvement of a mouse mammary tumor virus (MMTV) homologue in human breast cancer: Evidence for, against and possible causes of controversies. Microb. Pathog..

[2] World Health Organisation (2018). Latest global cancer data. Int. Agency Res. Cancer.

[3] Mackey J.R., Martin M., Pienkowski T., Rolski J., Guastalla J.P., Sami A., Glaspy J., Juhos E., Wardley A., Fornander T. (2013). Adjuvant docetaxel, doxorubicin, and cyclophosphamide in node-positive breast cancer: 10-year follow-up of the phase 3 randomised BCIRG 001 trial. Lancet Oncol..

[4] Carvalho C., Santos R., Cardoso S., Correia S., Oliveira P., Santos M., Moreira P. (2009). Doxorubicin: The good, the bad and the ugly effect. Curr. Med. Chem..

[5] Wefel J.S., Saleeba A.K., Buzdar A.U., Meyers C.A. (2010). Acute and late onset cognitive dysfunction associated with chemotherapy in women with breast cancer. Cancer.

[6] Brezden C.B., Phillips K.-A., Abdolell M., Bunston T., Tannock I.F. (2000). Cognitive function in breast cancer patients receiving adjuvant chemotherapy. J. Clin. Oncol..

[7] Schagen S.B., Van Dam F.S.A.M., Muller M.J., Boogerd W., Lindeboom J., Bruning P.F. (1999). Cognitive deficits after postoperative adjuvant chemotherapy for breast carcinoma. Cancer.

[8] Shilling V., Jenkins V., Morris R., Deutsch G., Bloomfield D. (2005). The effects of adjuvant chemotherapy on cognition in women with breast cancer—Preliminary results of an observational longitudinal study. Breast.

[9] Jansen C.E., Dodd M.J., Miaskowski C.A., Dowling G.A., Kramer J. (2008). Preliminary results of a longitudinal study of changes in cognitive function in breast cancer patients undergoing chemotherapy with doxorubicin and cyclophosphamide. Psychooncology.

[10] Orchard T.S., Gaudier-Diaz M.M., Phuwamongkolwiwat-Chu P., Andridge R., Lustberg M.B., Bomser J., Cole R.M., Belury M.A., Devries A.C. (2018). Low sucrose, omega-3 enriched diet has region-specific effects on neuroinflammation and synaptic function markers in a mouse model of doxorubicin-based chemotherapy. Nutrients.

[11] Tangpong J., Cole M.P., Sultana R., Joshi G., Estus S., Vore M., St. Clair W., Ratanachaiyavong S., St. Clair D.K., Butterfield D.A. (2006). Adriamycin-induced, TNF-α-mediated central nervous system toxicity. Neurobiol. Dis..

[12] Bigotte L., Olsson Y. (2004). Cytofluorescence localization of adriamycin in the nervous system. Acta Neuropathol..

[13] Seruga B., Zhang H., Bernstein L.J., Tannock I.F. (2008). Cytokines and their relationship to the symptoms and outcome of cancer. Nat. Rev. Cancer.

[14] Tangpong J., Cole M.P., Sultana R., Estus S., Vore M., St. Clair W., Ratanachaiyavong S., St. Clair D.K., Butterfield D.A. (2007). Adriamycin-mediated nitration of manganese superoxide dismutase in the central nervous system: Insight into the mechanism of chemobrain. J. Neurochem..

[15] Kirkpatrick L., Brady S. (2008). Basic Neurochemistry: Molecular, Cellular and Medical Aspects.

[16] Serhan C.N. (2014). Pro-resolving lipid mediators are leads for resolution physiology. Nature.

[17] Finocchiaro C., Segre O., Fadda M., Monge T., Scigliano M., Schena M., Tinivella M., Tiozzo E., Catalano M.G., Pugliese M. (2012). Effect of n-3 fatty acids on patients with advanced lung cancer: A double-blind, placebo-controlled study. Br. J. Nutr..

[18] Alfano C.M., Imayama I., Neuhouser M.L., Kiecolt-Glaser J.K., Smith A.W., Meeske K., McTiernan A., Bernstein L., Baumgartner K.B., Ulrich C.M. (2012). Fatigue, inflammation, and -γ3 and γ-6 fatty acid intake among breast cancer survivors. J. Clin. Oncol..

[19] American Cancer Society (2019). Cancer Treatment & Survivorship Facts & Figures 2016-2017.

[20] Villa A., Rizzi N., Vegeto E., Ciana P., Maggi A. (2015). Estrogen accelerates the resolution of inflammation in macrophagic cells. Sci. Rep..

[21] Loiola R.A., Wickstead E.S., Solito E., McArthur S. (2019). Estrogen promotes pro-resolving microglial behavior and phagocytic cell clearance through the actions of annexin A1. Front. Endocrinol..

[22] Gubbels Bupp M.R. (2015). Sex, the aging immune system, and chronic disease. Cell. Immunol..

[23] Lustberg M.B., Orchard T.S., Reinbolt R., Andridge R., Pan X., Belury M., Cole R., Logan A., Layman R., Ramaswamy B. (2018). Randomized placebo-controlled pilot trial of omega 3 fatty acids for prevention of aromatase inhibitor-induced musculoskeletal pain. Breast Cancer Res. Treat..

[24] Reagan-Shaw S., Nihal M., Ahmad N. (2007). Dose translation from animal to human studies revisited. FASEB J..

[25] Colas R.A., Shinohara M., Dalli J., Chiang N., Serhan C.N. (2014). Identification and signature profiles for pro-resolving and inflammatory lipid mediators in human tissue. Am. J. Physiol. Physiol..

[26] R Core Team (2013). A Language and Environment for Statistical Computing.

[27] Ash M., Chang A., Heiberger R., Kerns G.J., Lancelot R., Lesnoff M., Muenchen R., Murdoch D., Neuwirth E., Putler D. Package ‘ Rcmdr ’.

[28] Bates D., Maechler M., Bolker B. lme4: Linear Mixed-Effects Models Using S4 Classes (R Package Version 0.999999-0).

[29] Bozek K., Wei Y., Yan Z., Liu X., Xiong J., Sugimoto M., Tomita M., Pääbo S., Sherwood C.C., Hof P.R. (2015). Organization and evolution of brain lipidome revealed by large-scale analysis of human, chimpanzee, macaque, and mouse tissues. Neuron.

[30] Xia J., Psychogios N., Young N., Wishart D.S. (2009). MetaboAnalyst: A web server for metabolomic data analysis and interpretation. Nucleic Acids Res..

[31] Tsugawa H., Cajka T., Kind T., Ma Y., Higgins B., Ikeda K., Kanazawa M., Vandergheynst J., Fiehn O., Arita M. (2015). MS-DIAL: Data-independent MS/MS deconvolution for comprehensive metabolome analysis. Nat. Methods.

[32] Tsugawa H., Kind T., Nakabayashi R., Yukihira D., Tanaka W., Cajka T., Saito K., Fiehn O., Arita M. (2016). Hydrogen rearrangement rules: Computational MS/MS fragmentation and structure elucidation using MS-FINDER Software. Anal. Chem..

[33] Sumner L.W., Lei Z., Nikolau B.J., Saito K., Roessner U., Trengove R. (2014). Proposed quantitative and alphanumeric metabolite identification metrics. Metabolomics.

[34] Ikuo I., Katsumi I., Michihiro S. (1987). Absorption and transport of base moieties of phosphatidylcholine and phosphatidylethanolamine in rats. Biochim. Biophys. Acta (BBA)/Lipids Lipid Metab..

[35] Andersen P., Morris R., Amaral D., Bliss T., O’Keefe J. (2007). The Hippocampus Book.

[36] Basil M.C., Levy B.D. (2016). Specialized pro-resolving mediators: Endogenous regulators of infection and inflammation. Nat. Rev. Immunol..

[37] Wang X., Puerta E., Cedazo-Minguez A., Hjorth E., Schultzberg M. (2014). Insufficient Resolution response in the hippocampus of a senescence-accelerated mouse model—SAMP8. J. Mol. Neurosci..

[38] Hopperton K.E., Trépanier M.O., James N.C.E., Chouinard-Watkins R., Bazinet R.P. (2018). Fish oil feeding attenuates neuroinflammatory gene expression without concomitant changes in brain eicosanoids and docosanoids in a mouse model of Alzheimer’s disease. Brain. Behav. Immun..

[39] Trépanier M.O., Hopperton K.E., Giuliano V., Masoodi M., Bazinet R.P. (2018). Increased brain docosahexaenoic acid has no effect on the resolution of neuroinflammation following intracerebroventricular lipopolysaccharide injection. Neurochem. Int..

[40] Orr S.K., Palumbo S., Bosetti F., Mount H.T., Kang J.X., Greenwood C.E., Ma D.W.L., Serhan C.N., Bazinet R.P. (2013). Unesterified docosahexaenoic acid is protective in neuroinflammation. J. Neurochem..

[41] Belayev L., Khoutorova L., Atkins K.D., Eady T.N., Hong S., Lu Y., Obenaus A., Bazan N.G. (2011). Docosahexaenoic acid therapy of experimental ischemic stroke. Transl. Stroke Res..

[42] Marcheselli V.L., Hong S., Lukiw W.J., Tian X.H., Gronert K., Musto A., Hardy M., Gimenez J.M., Chiang N., Serhan C.N. (2003). Novel docosanoids inhibit brain ischemia-reperfusion-mediated leukocyte infiltration and pro-inflammatory gene expression. J. Biol. Chem..

[43] Levy B.D., Clish C.B., Schmidt B., Gronert K., Serhan C.N. (2001). Lipid mediator class switching during acute inflammation: Signals in resolution. Nat. Immunol..

[44] Chavko M., Nemoto E.M., Melick J.A. (1993). Regional lipid composition in the rat brain. Mol. Chem. Neuropathol..

[45] Miranda A.M., Bravo F.V., Chan R.B., Sousa N., Di Paolo G., Oliveira T.G. (2019). Differential lipid composition and regulation along the hippocampal longitudinal axis. Transl. Psychiatry.

[46] Mu Y., Gage F.H. (2011). Adult hippocampal neurogenesis and its role in Alzheimer’s disease. Mol. Neurodegener..

[47] Kuruba R., Hattiangady B., Shetty A.K. (2009). Hippocampal neurogenesis and neural stem cells in temporal lobe epilepsy. Epilepsy Behav..

[48] Harrison P.J. (2004). The hippocampus in schizophrenia: A review of the neuropathological evidence and its pathophysiological implications. Psychopharmacology.

[49] Cunnane S.C., Schneider J.A., Tangney C., Tremblay-Mercier J., Fortier M., Bennett D.A., Morris M.C. (2012). Plasma and brain fatty acid profiles in mild cognitive impairment and alzheimer’s disease. J. Alzheimer’s Dis..

[50] Manicke N.E., Wiseman J.M., Ifa D.R., Cooks R.G. (2008). Desorption electrospray ionization (DESI) mass spectrometry and tandem mass spectrometry (MS/MS) of phospholipids and sphingolipids: Ionization, adduct formation, and fragmentation. J. Am. Soc. Mass Spectrom..

[51] Fan Y., Meng H.M., Hu G.R., Li F.L. (2018). Biosynthesis of nervonic acid and perspectives for its production by microalgae and other microorganisms. Appl. Microbiol. Biotechnol..

[52] Martínez M., Mougan I. (2002). Fatty acid composition of human brain phospholipids during normal development. J. Neurochem..

[53] Astarita G., Jung K.M., Vasilevko V., DiPatrizio N.V., Martin S.K., Cribbs D.H., Head E., Cotman C.W., Piomelli D. (2011). Elevated stearoyl-CoA desaturase in brains of patients with Alzheimer’s disease. PLoS ONE.

[54] Nasaruddin M.L., Hölscher C., Kehoe P., Graham S.F., Green B.D. (2016). Wide-ranging alterations in the brain fatty acid complement of subjects with late Alzheimer’s disease as detected by GC-MS. Am. J. Transl. Res..

[55] Nasaruddin M.L., Pan X., McGuinness B., Passmore P., Kehoe P.G., Hölscher C., Graham S.F., Green B.D. (2018). Evidence that parietal lobe fatty acids may be more profoundly affected in moderate alzheimer’s disease (AD) pathology than in severe AD pathology. Metabolites.

[56] Iuliano L., Pacelli A., Ciacciarelli M., Zerbinati C., Fagioli S., Piras F., Orfei M.D., Bossù P., Pazzelli F., Serviddio G. (2013). Plasma fatty acid lipidomics in amnestic mild cognitive impairment and Alzheimer’s disease. J. Alzheimer’s Dis..

[57] Kageyama Y., Kasahara T., Nakamura T., Hattori K., Deguchi Y., Tani M., Kuroda K., Yoshida S., Goto Y.I., Inoue K. (2018). Plasma nervonic acid is a potential biomarker for major depressive disorder: A pilot study. Int. J. Neuropsychopharmacol..

[58] Carmeli C., Donati A., Antille V., Viceic D., Ghika J., von Gunten A., Clarke S., Meuli R., Frackowiak R.S., Knyazeva M.G. (2013). Demyelination in mild cognitive impairment suggests progression path to Alzheimer’s disease. PLoS ONE.

[59] Kelly L., Grehan B., Della Chiesa A., O’Mara S.M., Downer E., Sahyoun G., Massey K.A., Nicolaou A., Lynch M.A. (2011). The polyunsaturated fatty acids, EPA and DPA exert a protective effect in the hippocampus of the aged rat. Neurobiol. Aging.

[60] Minogue A.M., Lynch A.M., Loane D.J., Herron C.E., Lynch M.A. (2007). Modulation of amyloid-β-induced and age-associated changes in rat hippocampus by eicosapentaenoic acid. J. Neurochem..

[61] Taneo J., Adachi T., Yoshida A., Takayasu K., Takahara K., Inaba K. (2015). Amyloid β oligomers induce interleukin-1β production in primary microglia in a cathepsin B- and reactive oxygen species-dependent manner. Biochem. Biophys. Res. Commun..

[62] Miller E., Kaur G., Larsen A., Loh S.P., Linderborg K., Weisinger H.S., Turchini G.M., Cameron-Smith D., Sinclair A.J. (2013). A short-term n-3 DPA supplementation study in humans. Eur. J. Nutr..

[63] Cansev M., Wurtman R.J. (2007). Chronic administration of docosahexaenoic acid or eicosapentaenoic acid, but not arachidonic acid, alone or in combination with uridine, increases brain phosphatide and synaptic protein levels in gerbils. Neuroscience.

[64] Akbar M., Calderon F., Wen Z., Kim H.-Y. (2005). Docosahexaenoic acid: A positive modulator of Akt signaling in neuronal survival. Proc. Natl. Acad. Sci. USA.

[65] Ginsberg L., Rafique S., Xuereb J.H., Rapoport S.I., Gershfeld N.L. (1995). Disease and anatomic specificity of ethanolamine plasmalogen deficiency in Alzheimer’s disease brain. Brain Res..

[66] Wood P.L., Barnette B.L., Kaye J.A., Quinn J.F., Woltjer R.L. (2015). Non-targeted lipidomics of CSF and frontal cortex grey and white matter in control, mild cognitive impairment, and Alzheimer’s disease subjects. Acta Neuropsychiatr..

[67] Yamashita S., Kiko T., Fujiwara H., Hashimoto M., Nakagawa K., Kinoshita M., Furukawa K., Arai H., Miyazawa T. (2016). Alterations in the levels of amyloid-β, phospholipid hydroperoxide, and plasmalogen in the blood of patients with Alzheimer’s disease: Possible interactions between amyloid-β and these lipids. J. Alzheimer’s Dis..

[68] Fujino T., Yamada T., Asada T., Tsuboi Y., Wakana C., Mawatari S., Kono S. (2017). Efficacy and blood plasmalogen changes by oral administration of plasmalogen in patients with mild Alzheimer’s disease and mild cognitive impairment: A multicenter, randomized, double-blind, placebo-controlled trial. EBioMedicine.

[69] Han X., Holtzman D.M., McKeel D.W. (2001). Plasmalogen deficiency in early Alzheimer’s disease subjects and in animal models: Molecular characterization using electrospray ionization mass spectrometry. J. Neurochem..

[70] Chan R.B., Oliveira T.G., Cortes E.P., Honig L.S., Duff K.E., Small S.A., Wenk M.R., Shui G., Di Paolo G. (2012). Comparative lipidomic analysis of mouse and human brain with Alzheimer disease. J. Biol. Chem..

