# Supplementary material for: The Effects of Doxorubicin-based Chemotherapy and Omega-3 Supplementation on Mouse Brain Lipids
Source: Metabolites. 2019 Sep 29;9(10):208. doi: 10.3390/metabo9100208 (PMC6835930; doi:10.3390/metabo9100208)
Supplement: Supplementary file 1 [file metabolites-09-00208-s001.pdf]

Online Supplementary Material

The Effects of Doxorubicin-based Chemotherapy and Omega-3 Supplementation on the Mouse Brain Lipidome

Djawed Bennouna, Melissa Solano, Tonya S. Orchard, A. Courtney DeVries, Maryam Lustberg, Rachel E. Kopec

The online supplementary information provides additional details regarding the standards analyzed to unequivocally identify lipid species (**Table S1**), qualitative and quantitative MRMs transitions for SPM analysis (**Table S2**), the total ion chromatogram of brain extract in negative mode (**Figure S1**), the PCA of raw data including pooled QCs (**Figure S2**), Two-dimensional PCA plot, with datapoints representing all treated groups (**Figure S3**), Two-dimensional PCA plot, with datapoints representing individual animals treated with chemotherapy (**Figure S4**), Two-dimensional PCA plot, with datapoints representing individual animals supplemented with omega-3 (**Figure 5**), the fragmentation pattern of the detected phospholipids (**Figures S6-S11**), Heatmap of all biomarkers (**Figure 12**) and the biosynthetic pathway of omega-9 monounsaturated fatty acid synthesis (**Figure S13**).

Table S1: List of purified fatty acids used for fatty acid identification

CAS Number	Compound Name (synonyms)	Supplier
90175	13(*Z*)-docosenoic acid (erucic acid)	Cayman Chemical
13940	nervonic acid	Cayman Chemical
26169	13(*Z*)-eicosenoic acid (paullinic acid)	Cayman Chemical
90150	linoleic acid	Cayman Chemical
10010188	1-hexadecyl-lysophosphatidic acid	Cayman Chemical
90165	docosapentaenoic acid	Cayman Chemical
90110	eicosapentaenoic acid	Cayman Chemical
90330	11(*Z*),14(*Z*)-eicosadienoic acid	Cayman Chemical
90310	docosahexaenoic acid	Cayman Chemical
90190	5(*Z*),8(*Z*),11(*Z*)-eicosatrienoic acid (mead acid)	Cayman Chemical
16878	bovine phosphatidylethanolamine mixture	Cayman Chemical
20606	11(*Z*)-eicosenoic acid (gadolenic acid)	Cayman Chemical
sc-474901	22-tricosenoic acid	Santa Cruz Biotechnology
840499C	16:0-20:4 PG	Avanti
850144P	18:0-20:4 PI	Avanti

Table S2. Parameters for LC-MS/MS analysis of specialized pro-resolving mediators (SPMs) in negative mode

**Compound**	**Retention Time** (min)	**Precursor Ion Species**	**Precursor Ion** *m/z*	**Product Ions** *m/z* (Optimal Collision Energy, V)
RvE1	7.88	$[M-H]^-$	349.1	107.2 (10)[a], 195.1 (10)[b]
RvE1-d_4	7.82	$[M-H]^-$	353.2	109.2 (10)[a], 197.1 (10)[b]
RvD2	10.13,10.27[c]	$[M-H]^-$	375.12	141.1 (10)[b], 215.2 (10)[a]
RvD2-d_5	10.26	$[M-H]^-$	380.2	141.4 (10)[a], 175.1 (10)[b]
RvD3	10.27	$[M-H]^-$	375.12	115.1 (10)[a], 147.2 (10)[b]
RvD3-d_5	10.23	$[M-H]^-$	380.2	147.2 (10)[a], 152.2 (10)[b]
RvD1	10.65	$[M-H]^-$	375.12	121.2 (25)[a], 141.2 (10)[b]
RvD1-d_5	10.63	$[M-H]^-$	380.2	141.1 (10)[b], 220.2 (10)[a]
PD1	12.55	$[M-H]^-$	359.12	153.2 (10)[b], 206.2 (10)[a]
RvD5	12.60	$[M-H]^-$	359.12	199.3 (10)[b], 279.2 (10)[a]
MaR1	12.74	$[M-H]^-$	359.12	123.1 (10)[a], 250.1 (10)[b]
MaR1-d_5	12.71	$[M-H]^-$	364.2	123.2 (10)[b], 250.2 (10)[a]
LTB_4-d_4	12.93	$[M-H]^-$	339.2	197.2 (10)[a], 321.3 (10)[b]

[a] product ion used for identification

[b] product ion used for quantitation

[c] presumed isomer, observed at very low quantities in the standard

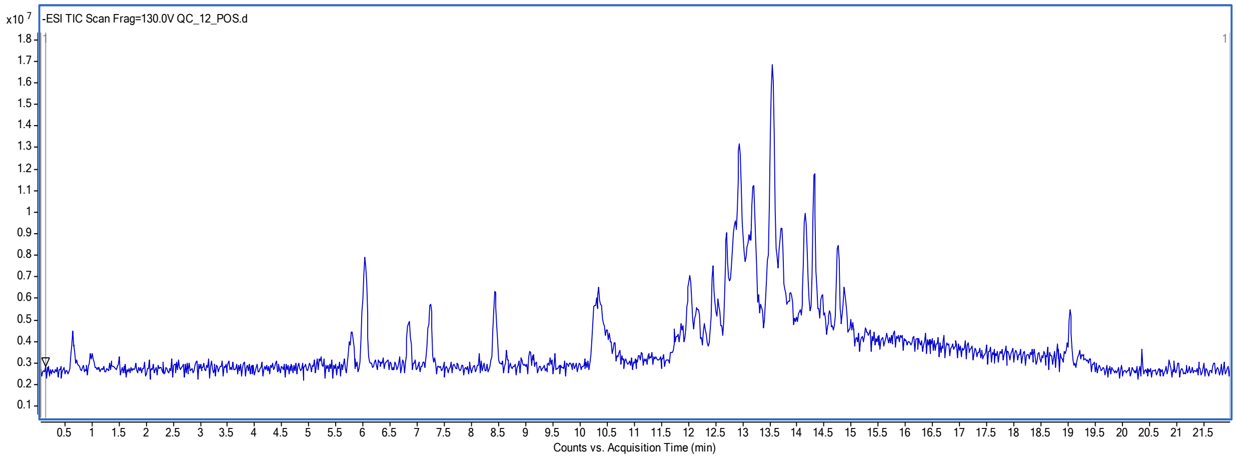

Figure S1. Total ion chromatogram (LC-MS) of hippocampus extract ionized in negative mode

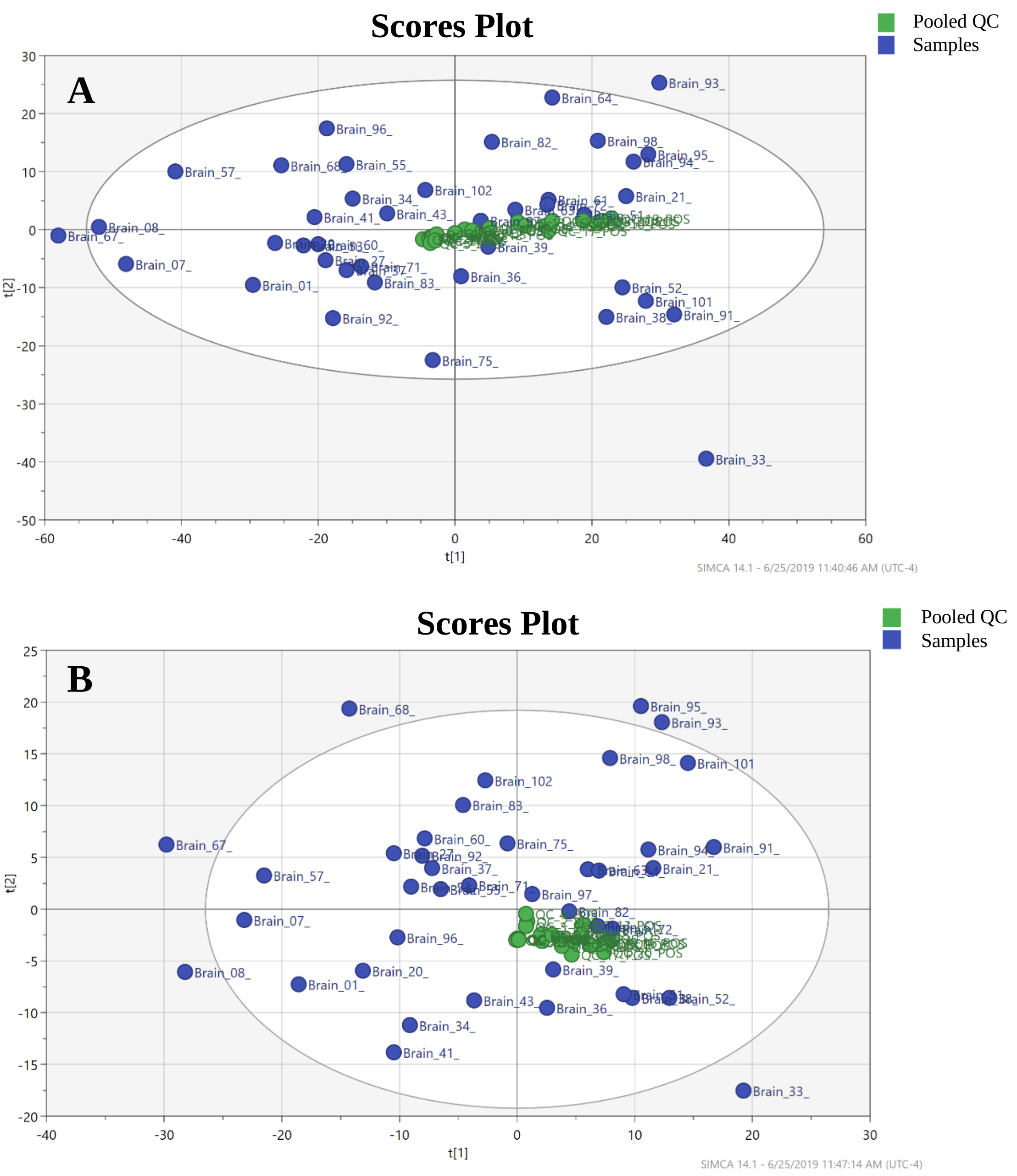

Figure S2. PCA scores plots depicting individual samples (blue dots), and pooled quality control (QC) samples (green dots) of (A) raw data before any filtration, and (B) filtered data (i.e. after removal of blank signals and false chromatographic peaks).

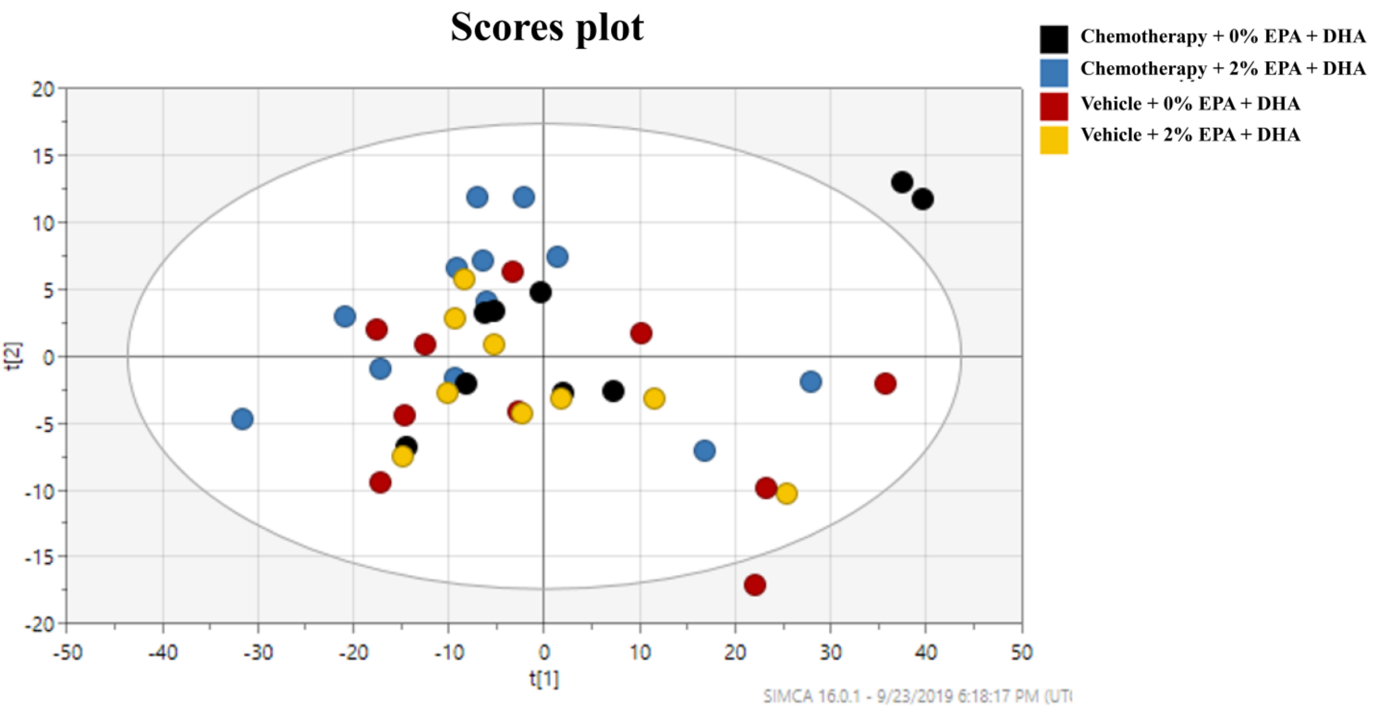

Figure S3. PCA scores plot showing all treated groups, with each datapoint representing individual samples. Animals treated with chemotherapy receiving 0% EPA+ DHA diet (black dots), animals treated with chemotherapy receiving 2% EPA+ DHA diet (blue dots), animals treated with vehicle receiving 0% EPA+ DHA diet (red dots), animals treated with vehicle receiving 2% EPA+ DHA diet (yellow dots) after raw metabolite intensities were $\log_{10}$ transformed and UV-scaled. PC1 explains 57.2% of the variance observed and PC2 explains 9.1% of the variance observed.

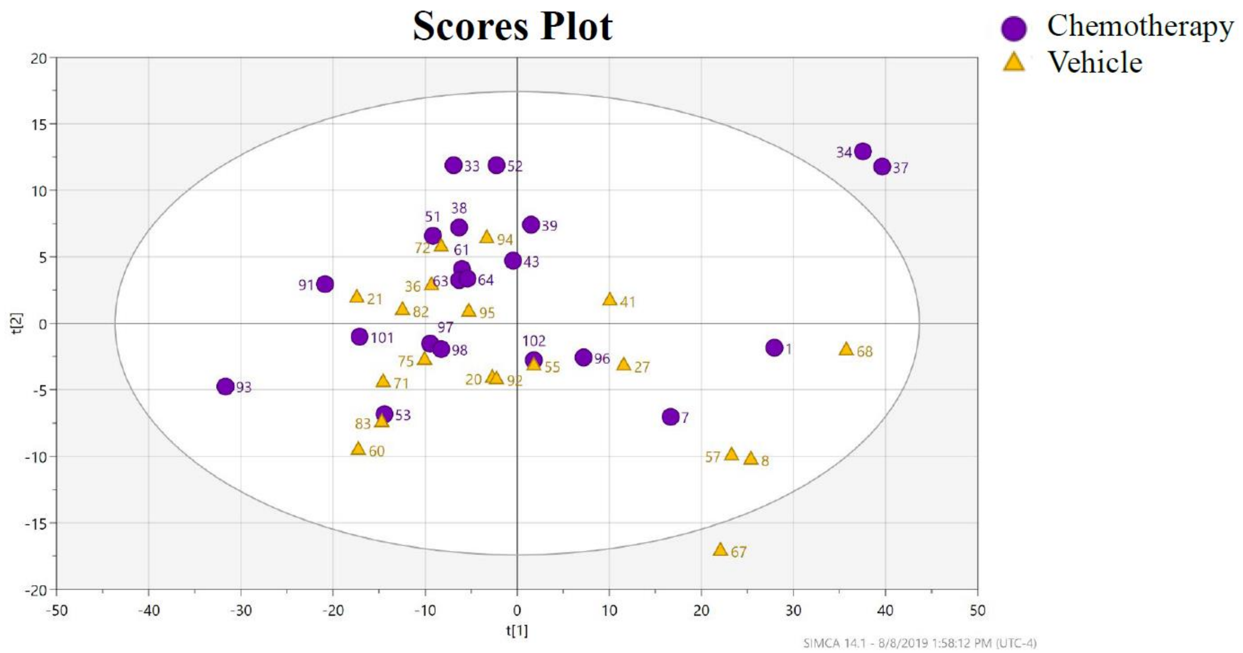

Figure S4. Two-dimensional PCA plot, with datapoints representing individual animals treated with chemotherapy (purple dot, n = 21) or vehicle (orange triangles, n = 19), after raw metabolite intensities were $\log_{10}$ transformed and UV scaled. PC1 explains 57.2% of the variance observed and PC2 explains 9.1% of the variance observed.

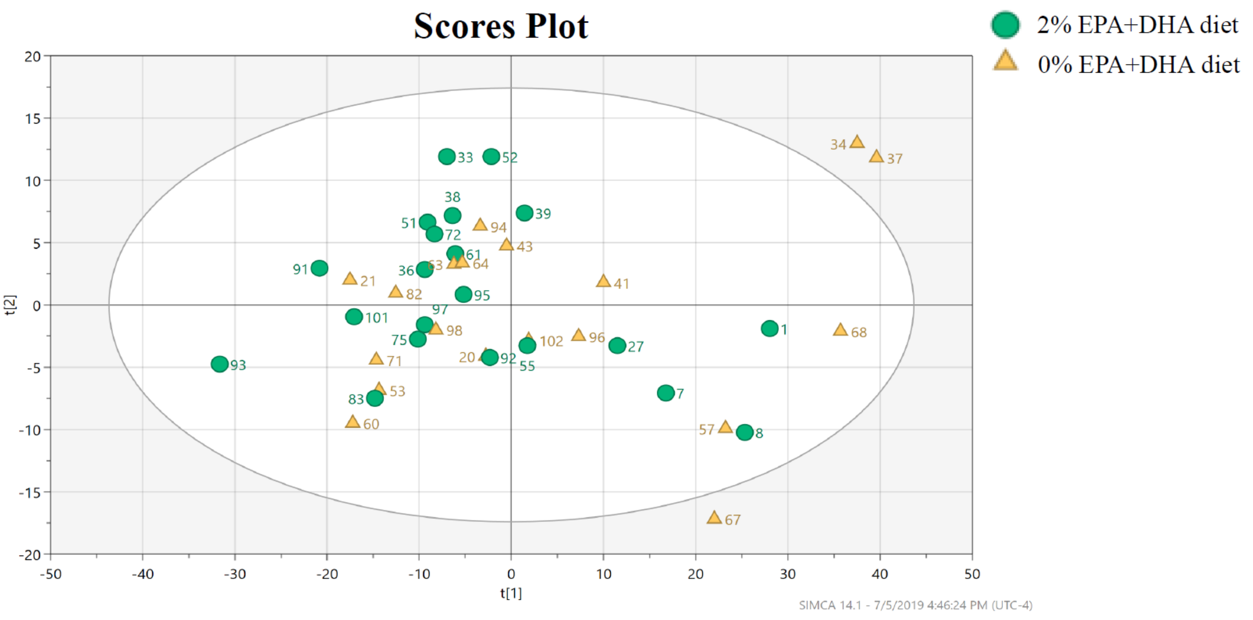

Figure S5. Two-dimensional PCA plot, with datapoints representing individual animals supplemented with 2% EPA+DHA diet (green dot, $n = 21$) or 0% EPA+DHA diet (orange triangles, $n = 19$), after raw metabolite intensities were $\log_{10}$ transformed and UV scaled. PC1 explains 57.2% of the variance observed and PC2 explains 9.1% of the variance observed.

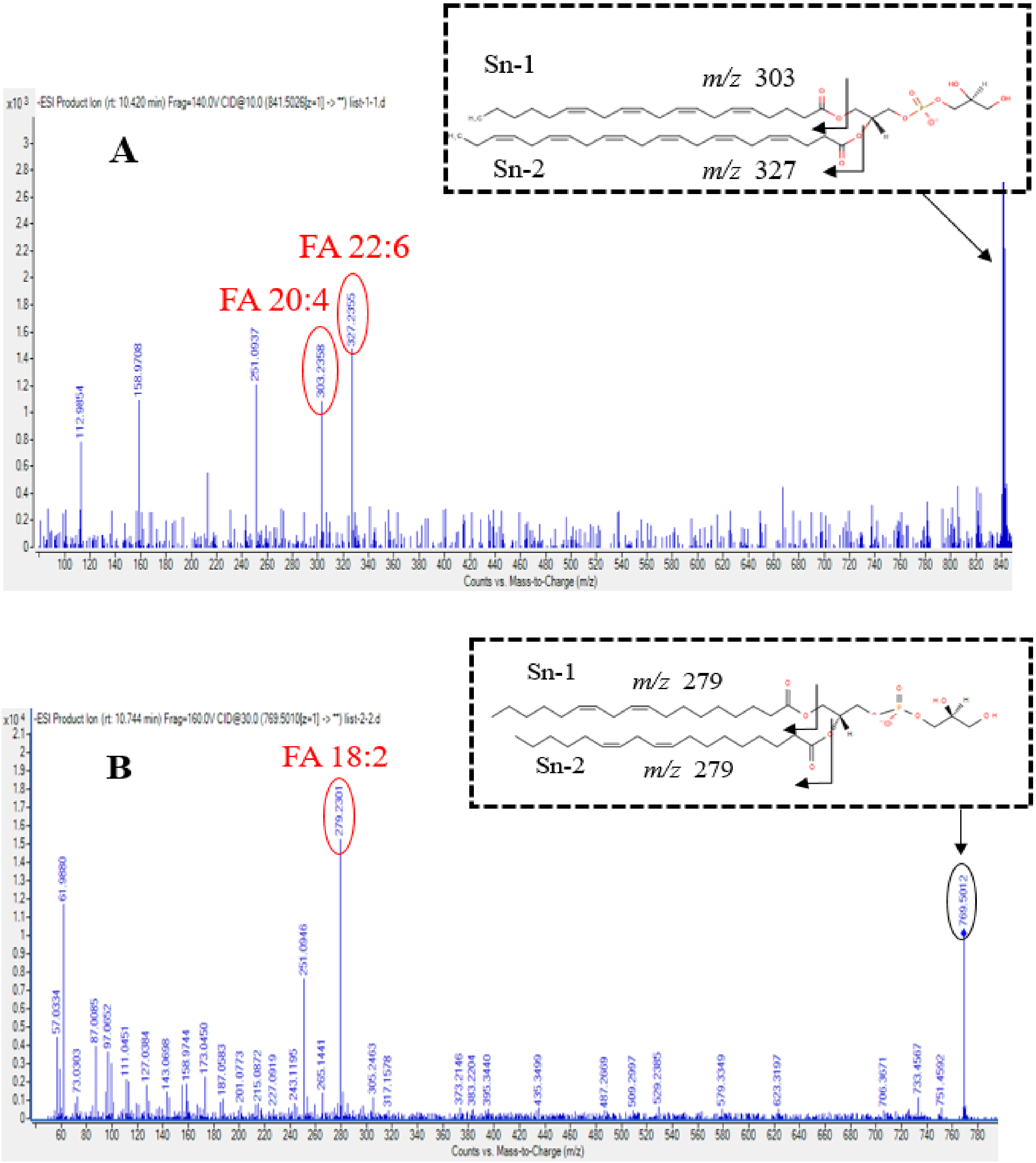

Figure S6. Product ion spectra of (**A**) PG (20:4/22:6) showing fragmentation of the precursor [M-H]$^-$ *m/z* 841 and (**B**) PG (18:2/18:2) showing fragmentation of the precursor [M-H]$^-$ at *m/z* 769. In panel **A**, carboxylate anion products at *m/z* 327 and 303 reflect the 20:4- and 22:6-acyl constituent losses, respectively. In panel **B**, the anion with *m/z* 279 reflects the presence of two 18:2-acyl residing at the sn-1 and sn-2 positions.

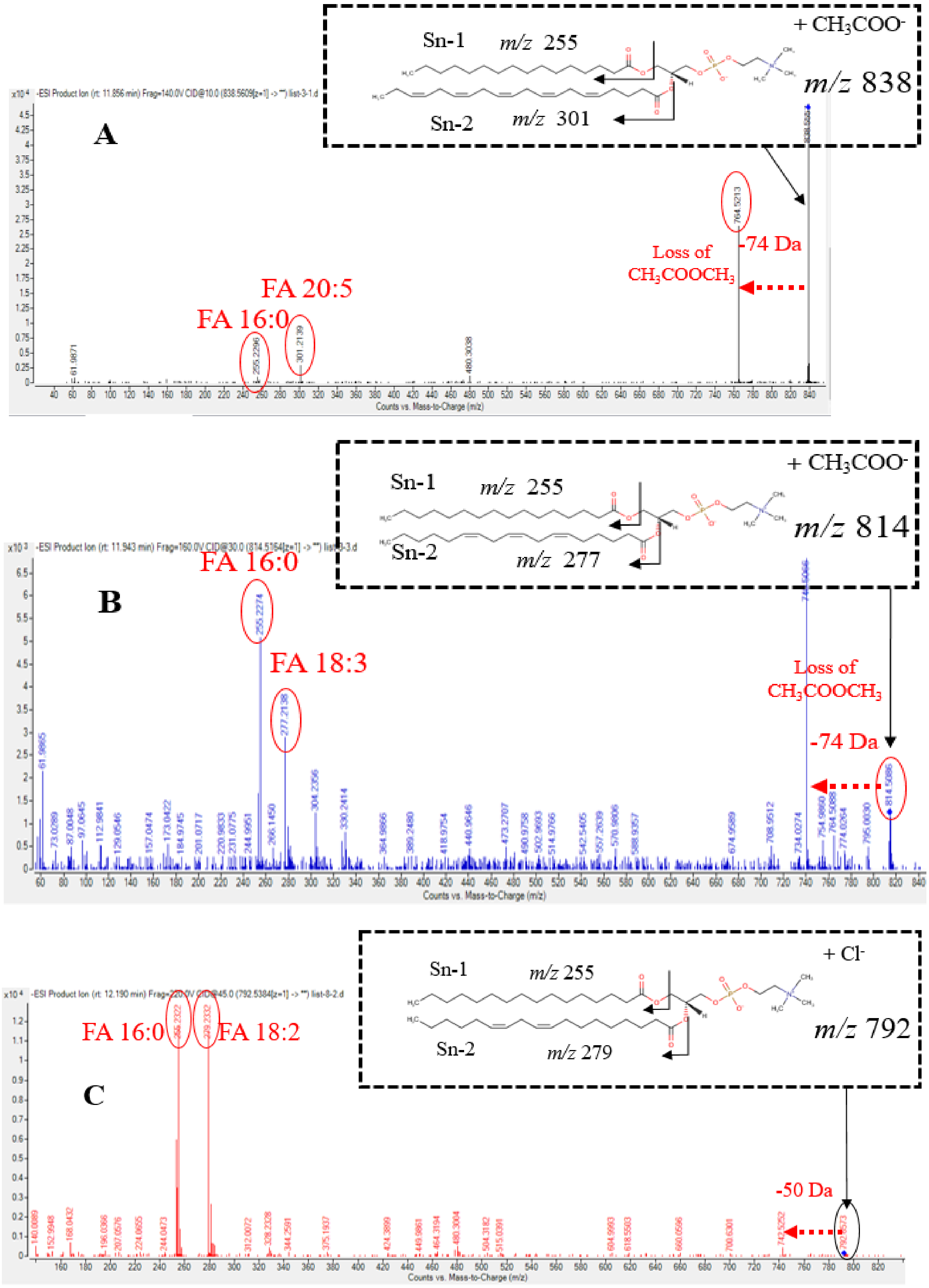

Figure S7. Product ion spectra of (**A**) PC (16:0/20:5) at *m/z* 838 corresponding to $[M+ CH_3COO]^-$, (**B**) PC (16:0/18:3) at *m/z* 814 corresponding to $[M+ CH_3COO]^-$ and (**C**) PC (16:0/18:2) at *m/z* 792 corresponding to $[M+Cl]^-$. The neutral loss of CH_3COOCH_3, observed in panels A and B, is known to be a diagnostic ion observed in negative mode for lipids with a choline functional group[1]. In panel **A**, product carboxylate anions at *m/z* 301 and 255, reflect the 16:0- and 20:5-acyl

constituents, respectively. In panel **B**, product carboxylate anions at *m/z* 277 and 255 reflect the 16:0- and 18:3-acyl constituents, respectively. In panel **C**, a neutral loss of 50 Da corresponded to a common neutral loss of the added Cl^- and a methyl group observed for lipids with a choline functional group[1]. This loss yields a fragment ion at *m/z* 742. Product carboxylate anions at *m/z* 279 and 255 reflect the loss of 16:0- and 18:2-acyl constituents, respectively.

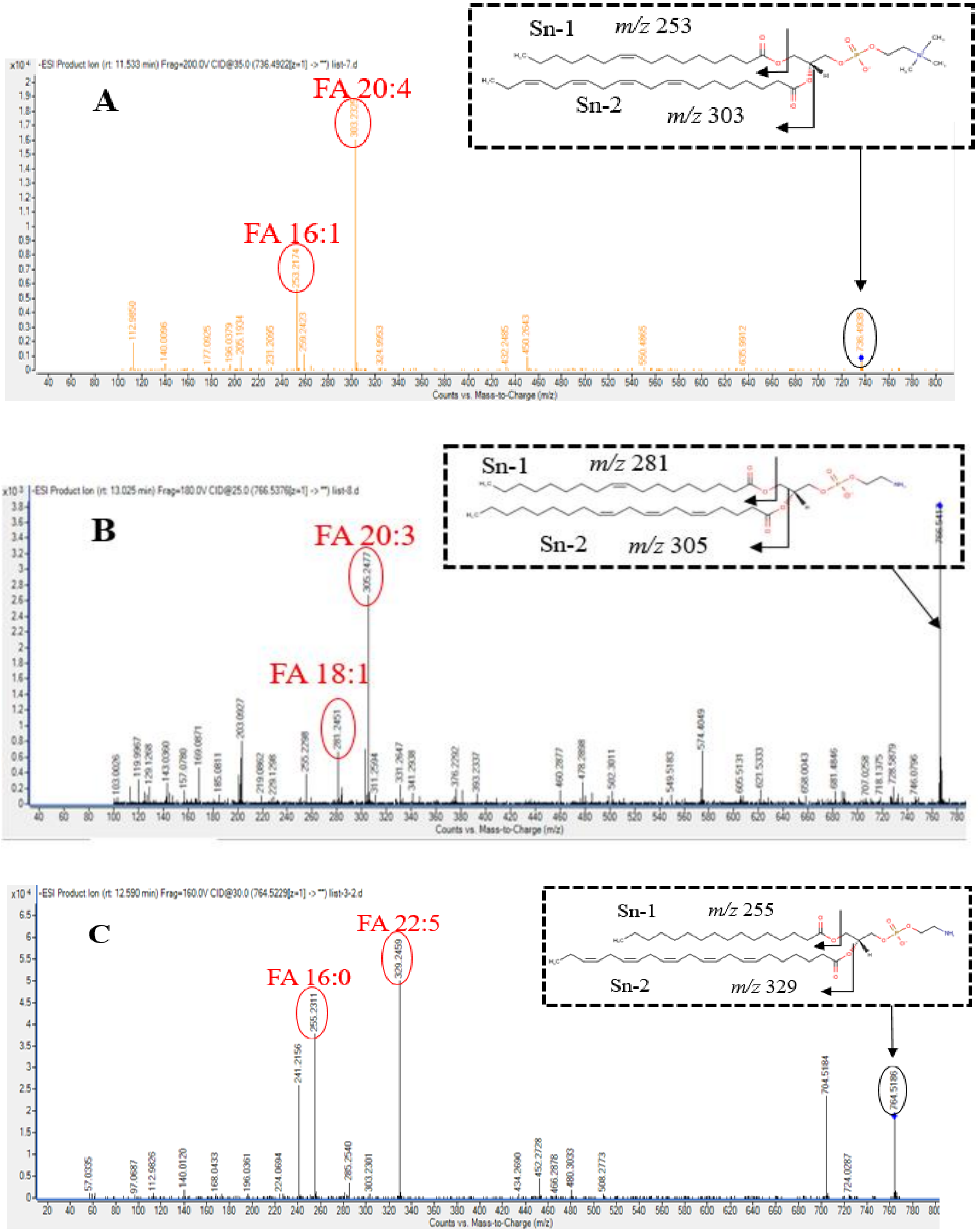

Figure S8. Product ion spectra of (**A**) PE (16:1/20:4) at *m/z* 736 corresponding to $[M-H]^-$, (**B**) PE (18:1/20:3) at *m/z* 766 corresponding to $[M-H]^-$, and (**C**) PE (16:0/22:5) at *m/z* 764 corresponding to $[M-H]^-$. In panel **A**, product carboxylate anions at m/z 303 and 253, reflect 16:1- and 20:4-acyl constituents respectively. In panel **B,** product carboxylate anions at m/z 305 and 281, correspond to 16:1- and 20:4-acyl constituents, respectively. In panel **C**, product carboxylate anions at *m/z* 329 and 255, reflect 18:1- and 20:3-acyl constituents, respectively.

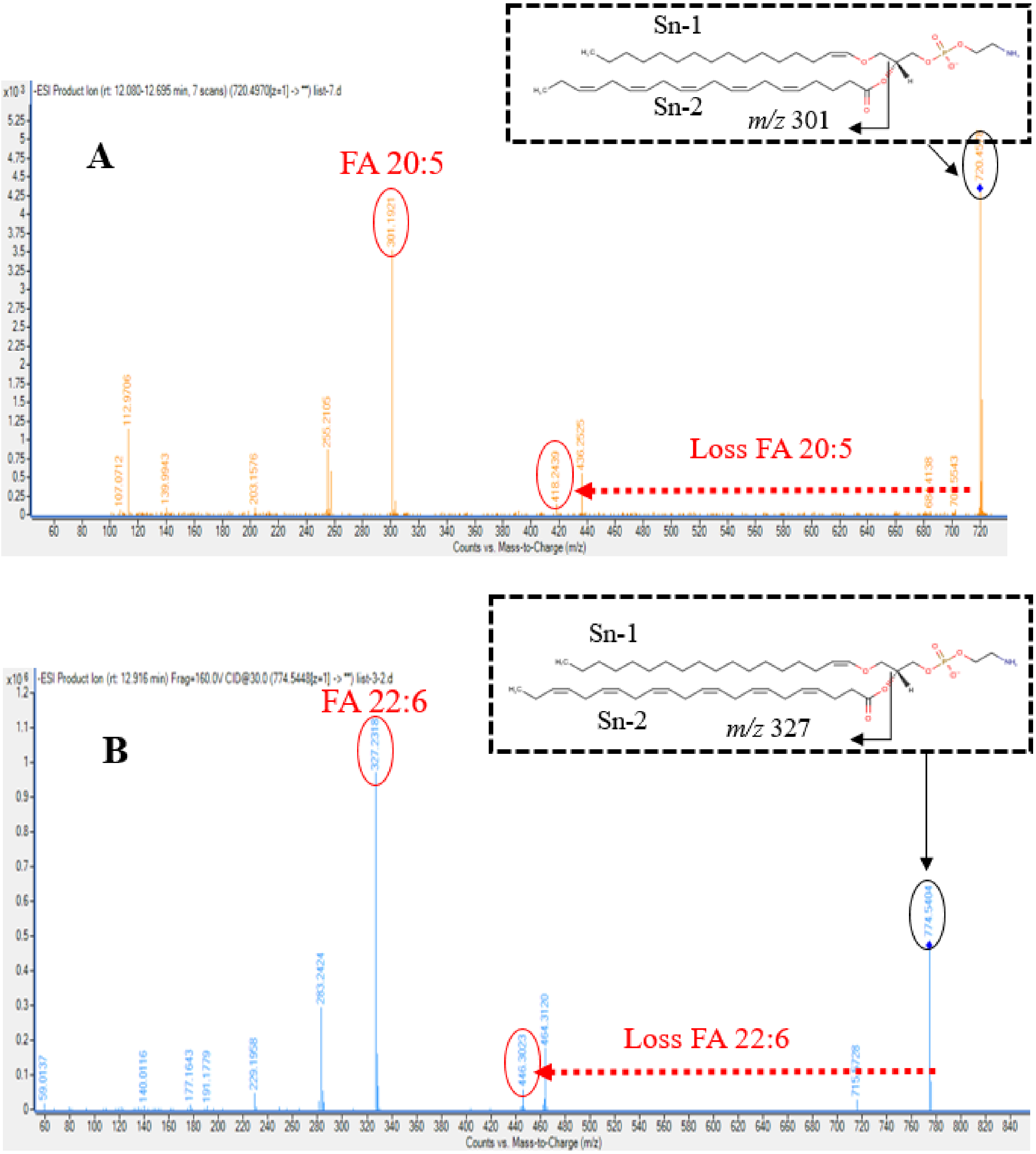

Figure S9. Product ion spectra of (**A**) plasmalogen PE(P-16:0/20:5) at *m/z* 736 corresponding to [M-H]$^-$, and (**B**) plasmalogen PE(P-18:0/22:6) at *m/z* 774, corresponding to [M-H]$^-$. In panel **A**, anions at *m/z* 301 and 418 reflect, a 20:5-acyl constituent and an anion (designated as [M-H-R2CO$_2$H]$^-$) resulting from loss of this same acyl group (20:5) at sn-2 as a ketene, respectively. In panel **B**, anions at *m/z* 327 and 446 correspond, to the acyl group at sn-2 (22:6) and an anion (designated as [M-H-R2CO$_2$H]$^-$) resulting from loss of this same acyl group (22:6) at sn-2 as a ketene, respectively. Note also that previous fragmentation studies of plasmalogen speciesobserved similar results in which little to no fragmentation occurs at the vinyl-ether bond at the sn-1 position[2].

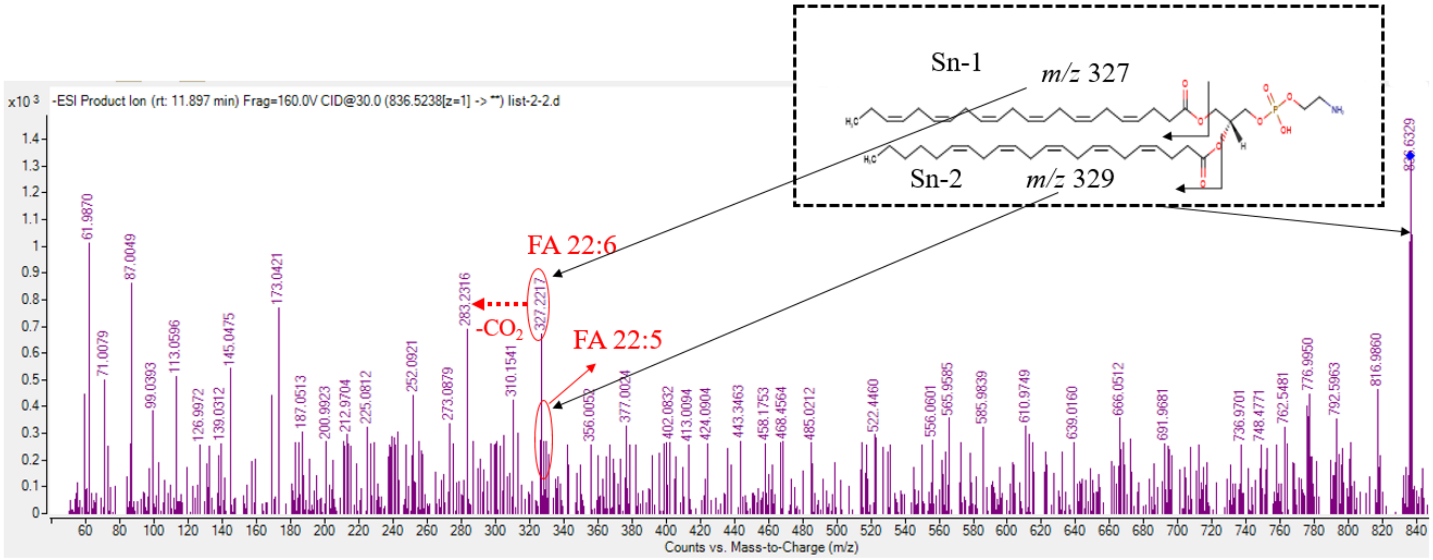

Figure S10. Product-ion spectra of PE (22:6/22:5). The product-ion spectrum of [M-H]- ion at m/z 836, contains carboxylate anions at m/z 327 and 329, which reflects 22:6- and 22:5-acyl constituents.

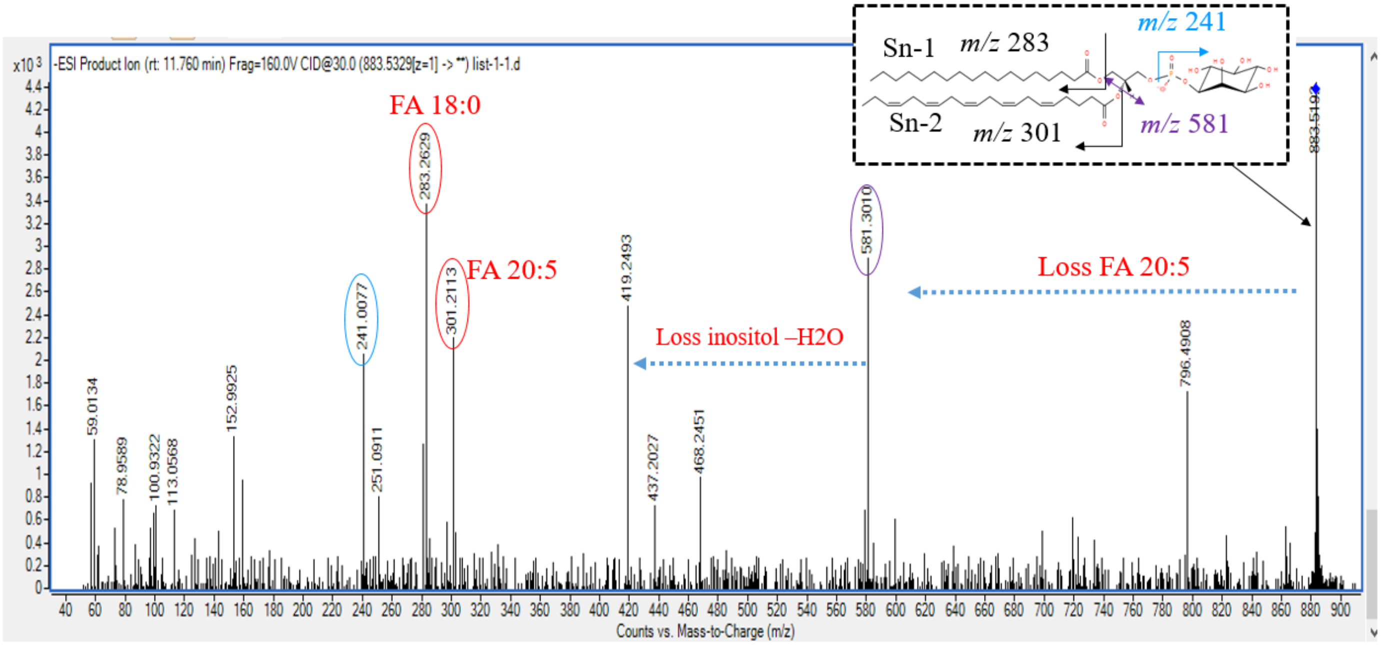

Figure S11. Product ion spectra of PI 18:0/20:5 at *m*/*z* 883, corresponding to [M-H]-. Carboxylate anions at m/z 301 and 283, reflect 20:5- and 18:0-acyl constituents, respectively.

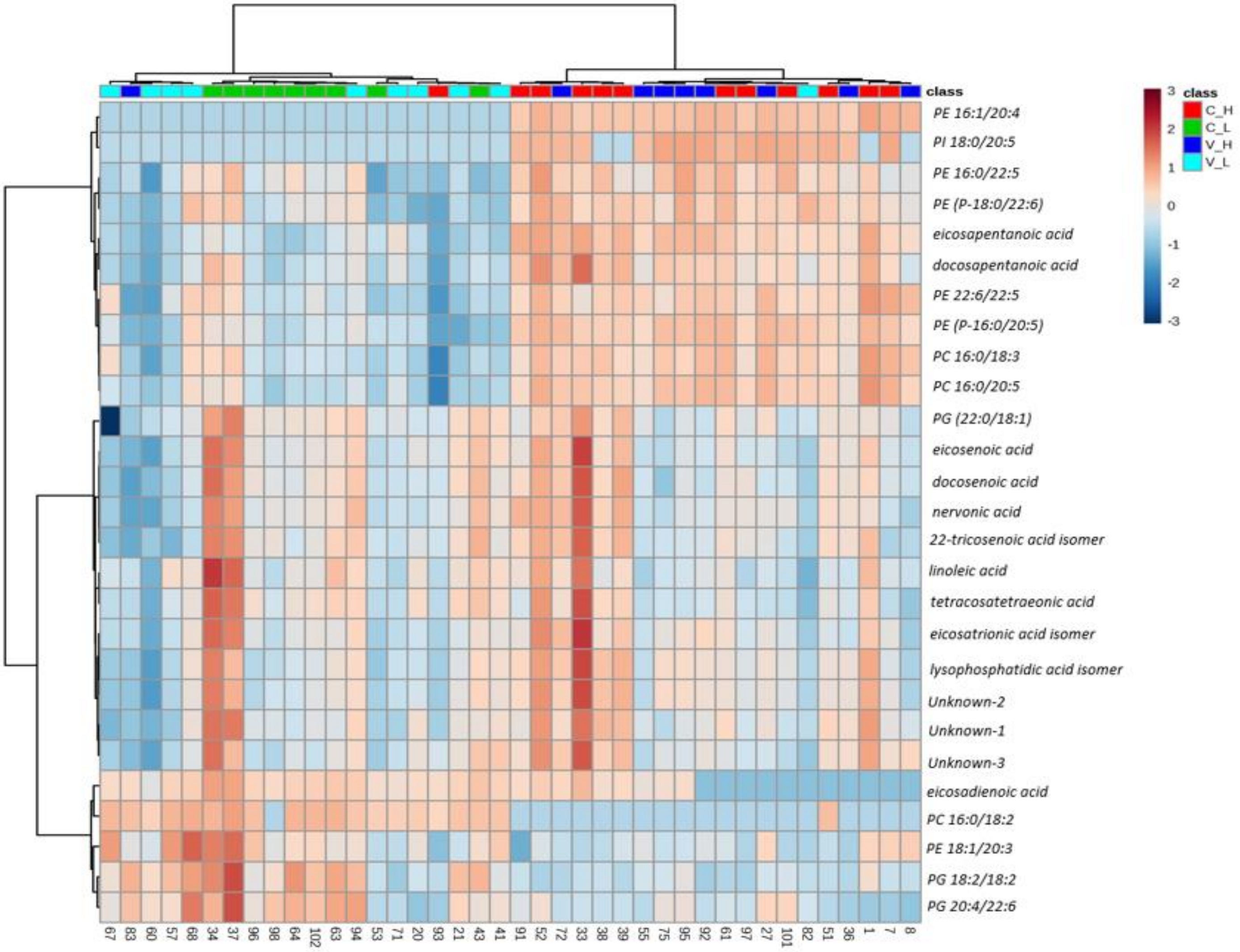

Figure S12. The heatmap is a graphical representation of data where the samples are clustered according to the proximity of the intensity of metabolites. Pearson distance and the Ward method were used for this heatmap. Each colored cell on the map corresponds to an intensity value from our data table, with samples in columns and compounds in rows.

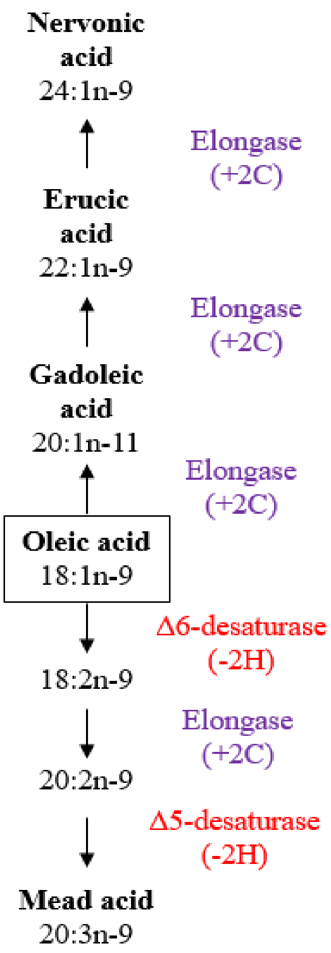

Figure S13. Omega-9 polyunsaturated fatty acid biosynthesis pathway[3]

References

(1) Pham, T. H.; Zaeem, M.; Fillier, T. A.; Nadeem, M.; Vidal, N. P.; Manful, C.; Cheema, S.; Cheema, M.; Thomas, R. H. Targeting Modified Lipids during Routine Lipidomics Analysis Using HILIC and C30 Reverse Phase Liquid Chromatography Coupled to Mass Spectrometry. *Sci. Rep.* **2019**, *9* 1–15.

(2) Jackson, S. N.; Wang, H. Y. J.; Woods, A. S. In Situ Structural Characterization of Glycerophospholipids and Sulfatides in Brain Tissue Using MALDI-MS/MS. *J. Am. Soc. Mass Spectrom.* **2007**, *18*, 17–26.

(3) Astarita, G.; Jung, K. M.; Vasilevko, V.; DiPatrizio, N. V.; Martin, S. K.; Cribbs, D. H.; Head, E.; Cotman, C. W.; Piomelli, D. Elevated Stearoyl-CoA Desaturase in Brains of Patients with Alzheimer's Disease. *PLoS One* **2011**, *6*, 1–9.
